# Comprehensive geriatric assessment, and related interventions, to improve outcomes for older patients undergoing transcatheter aortic valve implantation (TAVI): a systematic review

**DOI:** 10.1007/s41999-024-01035-5

**Published:** 2024-09-27

**Authors:** Katherine Chin, Rosalind Jones, Eleni Lester, Alice Hegarty, Lieze Thielemans, Rebekah Schiff

**Affiliations:** 1 Department of Ageing and Health, Guy’s and St Thomas’ NHS Foundation Trust, London, SE1 7EH UK; 2 King’s College London, London, WC2R 2LS UK

**Keywords:** Transcatheter aortic valve implantation (TAVI), Frailty, Older people, Comprehensive geriatric assessment (CGA), Cardiovascular disease

## Abstract

**Introduction:**

Transcatheter aortic valve implantation (TAVI) is a treatment for people with severe symptomatic aortic stenosis, particularly those living with frailty. Increasing frailty is associated with poorer outcomes post-TAVI. Comprehensive Geriatric Assessment (CGA) has been shown in other settings to improve outcomes in those with frailty, including perioperatively. This systematic review aims to determine whether CGA, or interventions targeting its components, improves outcomes for older people undergoing TAVI.

**Methods:**

EMBASE, MEDLINE, CINAHL and Cochrane CENTRAL were searched on 09/01/23 and then the search was rerun on the 16/04/24. The review was registered on PROSPERO (CRD42022299955). Included studies had to evaluate either CGA, or a single- or multi-domain intervention targeting components of CGA, in those aged ≥ 65.

**Results:**

From 4091 papers, 24 met the inclusion criteria. Two studies assessed CGA pre-TAVI and reported mixed improvements in functional independence but no change in length of stay or post-operative delirium, although both studies had a serious risk of bias. Fifteen papers described an exercise-based intervention, and 1 paper detailed a Cognitive Behavioural Therapy-based intervention. Seven studies evaluated a multi-component intervention. There were conflicting results reported for the multi-component and single-component interventions. All studies had at least a moderate risk of bias.

**Conclusion(s):**

There is a lack of evidence to determine whether CGA, or related interventions, improve outcomes for older adults undergoing-TAVI. The evidence for perioperative CGA, and the results of this review, support the need for well-designed trials evaluating whether CGA improves outcomes post-TAVI for older adults living with frailty.

**Supplementary Information:**

The online version contains supplementary material available at 10.1007/s41999-024-01035-5.

## Key summary points

**Aim:**

To review the evidence for using Comprehensive Geriatric Assessment, or related interventions, to improve outcomes for older patients undergoing Transcatheter Aortic Valve Implantation (TAVI).

**Findings:**

There lacks evidence for use of CGA, or related interventions, in older adults undergoing TAVI due to the low quality of studies.

**Message:**

Further research is required to investigate whether CGA improves outcomes for older adults undergoing TAVI.

**Supplementary Information:**

The online version contains supplementary material available at 10.1007/s41999-024-01035-5.

## Introduction

Transcatheter aortic valve implantation (TAVI) is a well-established treatment option for moderate to high-risk older adults with symptomatic severe aortic stenosis. If left untreated, aortic stenosis has a ~ 50% mortality rate within 2 years of symptom onset [1]. The number of TAVI procedures is increasing and is forecast to continue doing so across the UK, Europe, the United States and Canada [2]. Indeed, in 2022/23, 7,669 TAVI procedures took place in the UK, which is 13% higher than in 2021/22 [3]. The average age of the patient remains consistently around 80 years across European countries and the United States [4–7].

A fast-emerging area of interest is the role of frailty in this patient population, particularly as it has been shown to be a predictor of morbidity and mortality post-procedure [8, 9]. The presence of frailty, as measured by the Essential Frailty Toolset (EFT), was associated with a 3.3-times increased risk of mortality at 30 days post-procedure, and 3.7-times increased risk of mortality at 1 year [8]. The importance of frailty as a prognostic indicator is reflected by the incorporation of a frailty assessment into the 2017 American College of Cardiology guidelines [9, 10].

A recent review outlined the current evidence for targeting frailty in older adults with any cardiovascular disease (CVD) [11]. The authors described studies which trialled either physical, pharmacological, nutritional, cognitive, or psychosocial interventions. They concluded that multi-component interventions are required to manage frailty in CVD and acknowledge that future clinical trials would benefit from focusing on specific cardiac populations.

Comprehensive geriatric assessment (CGA) is a multi-dimensional holistic assessment that addresses an older person’s physical, psychological, functional, environmental and social health, and includes a medication review [12]. Crucially, it includes the formulation and enactment of an optimisation plan which addresses issues identified during the assessment [12, 13]. It is an established intervention shown to improve quality of life (QoL) and survival for older adults in hospital [13], and reduce frailty progression and health care utilisation in outpatient settings [14, 15]. Notably, it has been shown to successfully improve outcomes in cardiology wards and in the perioperative setting of vascular or hip-fracture surgery [15–17]. This includes reducing the length of hospital stay (LoS), mortality and incidence of complications post-procedure, and improved QoL and functional status.

Given the relationship between frailty and TAVI outcomes, CGA has face validity as a treatment to improve outcomes for those undergoing TAVI. This review investigates whether the evidence base supports the use of CGA, or interventions targeting its component domains, as a method to improve outcomes for older adults undergoing TAVI.

## Methods

A systematic review was performed in line with the PRISMA guidelines (Appendix 1) and registered on PROSPERO (CRD42022299955).

### Search strategy

A search of EMBASE, MEDLINE, CINAHL, and Cochrane CENTRAL was performed using the pre-specified search strategy (Appendix 2) on the 9th January 2023. The search was rerun on the 16th April 2024 to ensure the results were up to date. Search terms encompassed the categories of (i) older people, (ii) TAVI, (iii) assessment and interventions, (iv) study design.

Whilst developing the protocol, a preliminary search of the databases suggested that there would be limited evidence evaluating the effect of CGA in patients with frailty undergoing TAVI. Therefore, the scope was broadened to include studies evaluating interventions targeting multiple- or single-domains usually addressed as part of a CGA, including physical, psychological, functional, pharmacological, and socioeconomic domains [12]. This would potentially inform interventions that might form part of a CGA optimisation plan.

### Eligibility criteria

Studies were included if they (i) were randomised controlled trials (RCTs), non-randomised controlled trials, observational cohort studies or controlled before-and-after study designs; (ii) included patients who had undergone, or were planned to undergo, an elective TAVI, and were ≥ 65 years old (if age range not given, when > 97.5% of the sample were ≥ 65 years old according to standard deviation i.e. mean minus 1.96 × standard deviation) (iii) evaluated a pre- or post-procedure CGA or an intervention targeting a component domain of CGA, and aimed at improving outcomes following a TAVI. Studies were only considered to have implemented a CGA if the intervention included both the assessments of the key domains of physical, psychological, functional, and social health, and an enacted optimisation plan; (iv) measured at least one key outcome likely to be influenced by CGA and of importance to older adults and health care systems, namely functional independence, physical performance, QoL, nutritional status, cognitive status, mental health, mortality or LoS.

Given the absence of any studies targeted specifically at patients identified as living with frailty, this was not one of the inclusion criteria, and an age cut-off alone used.

The titles and abstracts of records were collated in Microsoft Excel and duplicates removed. They were independently screened for inclusion by two reviewers (RJ, EL), according to pre-specified criteria. When the search was rerun, the title and abstracts were reviewed by KC, AH, LT, GW and AP. Full texts were reviewed by KC, RJ and EL. Any discrepancies were resolved by a fourth reviewer, RS.

### Data extraction and analysis

Data were extracted by a sole reviewer (RJ) and then cross-checked by EL and KC. For each study, the authors extracted the study design, participant demographics, intervention characteristics, comparator type, and any measures of the key outcomes as specified in the eligibility criteria, and frailty assessments. Synthesis of the evidence base was performed via tabulation of key study features, categorised by type of intervention. Data were extracted in its original form without conversion.

Each paper was independently assessed for risk of bias by three reviewers, (RJ, EL, KC), using the domains specified by the Cochrane RoB (randomised studies) [18] and ROBINS-I tool [19] (non-randomised studies) in line with Cochrane handbook guidance, then cross-checked. Where there were a sufficient number of studies to facilitate the use of the GRADE criteria [20], the certainty of evidence was assessed in relation to mortality, functional independence and QoL. These outcomes were selected as the authors classified them as important to patients and, as a result, important for making decisions regarding changing clinical practice.

### Intervention categorisation

For the purpose of analysis, the included studies were grouped into: (i) CGA, (ii) multi-component interventions, (iii) single-component interventions. This was defined as:

i. *CGA*—a multi-component intervention addressing the physical, psychological, functional, and social health of an individual. Importantly, it needed to include both an assessment of all domains and a subsequent enacted tailored optimisation plan addressing the issues identified.
ii. *Multi-component interventions*—interventions which addressed multiple components but not all of the ones included in a CGA, or did not implement a tailored optimisation plan.
iii. *Single-component interventions*—interventions which addressed a single domain of CGA.

## Results

**Fig. 1:**
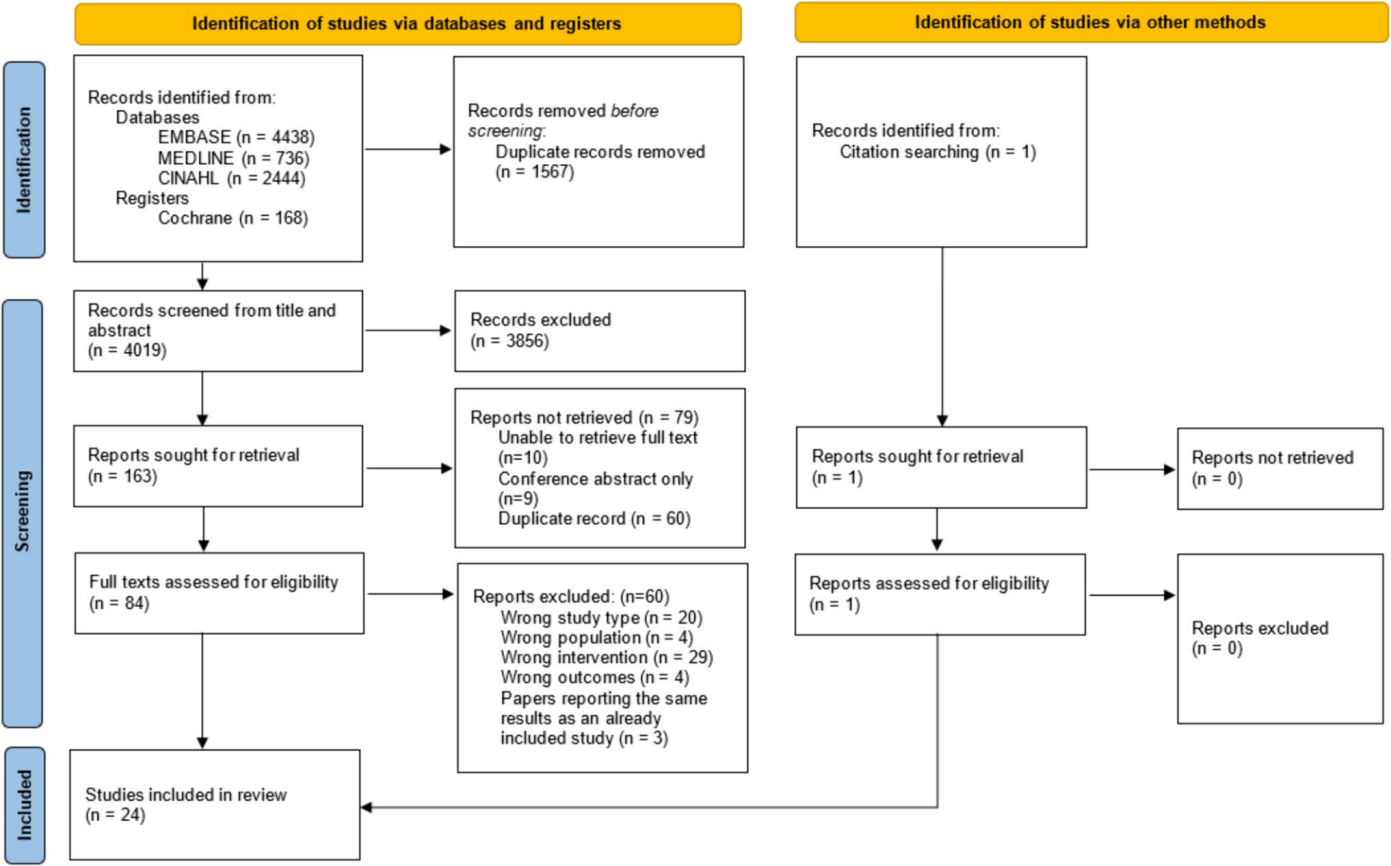
PRISMA flow diagram

Of the 4019 publications identified using the search terms, 24 met the inclusion criteria after full text screening (Fig. 1), which totalled 7068 patients undergoing TAVI. The reasons for exclusion at full text review are detailed in Appendix 3. Characteristics of the included studies are summarised in Table 1. Two studies delivered a CGA [21, 22], seven studies assessed the effect of a multi-component intervention [23–29], and 15 studies assessed a single-component intervention [30–44]. Of note, two studies were excluded at full text screening as one used CGA as a screening tool on which to base recommendations for whether a person was appropriate for a TAVI [45], and the other used CGA as a prognostic measure [46].

### Risk of bias in included studies

**Fig. 2:**
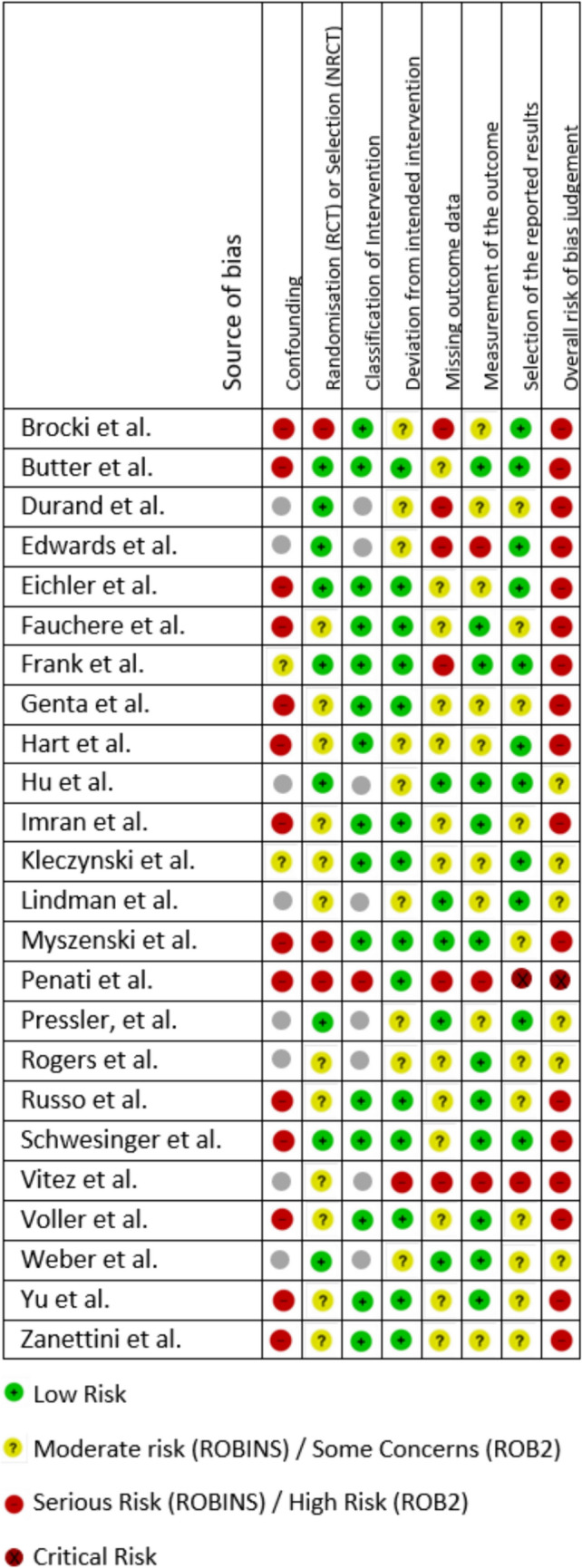
Risk of bias summary table

The risk of bias assessments are summarised in Fig. 2. Full details of the rationale behind each risk of bias judgement can be found in Appendix 4. A major source of bias was confounding. Thirteen papers described observational studies with inadequate controls. There is a known improvement of cardiovascular function following the correction of aortic stenosis, which will independently influence outcomes, such as QoL [47, 48]. This limitation was rarely mentioned in the studies [25, 28, 39, 41].

There was also judged to be a moderate risk of selection bias in some studies due to the lack of randomisation or provision of detail regarding the referral criteria for participation. One study [44], was found to have an overall critical risk of bias due to the risk of reporting bias as per the ROBINS-I tool. Therefore, it has been excluded from detailed synthesis.

### Certainty of evidence

Applying the GRADE criteria, there was very low-certainty evidence to suggest that multi-component or single-component interventions impacts QoL, functional independence, or mortality (Appendix 5). Similarly, there was very low-certainty evidence to support that CGA improves functional independence. The authors were not able to assess the certainty of evidence to suggest that CGA impacts QoL or mortality as these outcomes were not measured in this study [21, 22].

### Description of included studies

#### (i) CGA

One prospective observational study assessed the effect of CGA on post-TAVI outcomes [21]. Through a number of assessment scores (fried frailty scale, mini-mental state examination, Barthel index, hospital anxiety and depression scale, etc.) the pre-TAVI CGA evaluated the ability to complete self-care, cognitive function, nutritional status, anxiety and depression, frailty, and exercise capacity. The authors have not stated who conducted the CGA. A “tailor[ed]” intervention was formulated based on the results of the CGA assessment. The intervention was delivered in three phases: 1–3 days before the TAVI, within 24 h after TAVI, and then 1-month post-discharge. There was no control group and instead comparison was made with the pre-TAVI baseline measures. As a result, this study was assessed to be at a serious risk of bias overall.

The authors demonstrated a significant improvement in physical performance (*p* < 0.001), nutritional status (*p* = 0.001), cognition (*p* < 0.001), and hospital anxiety and depression scale (*p* = 0.001). There was also a significant improvement in the proportion of participants with frailty (*p* < 0.001) and in mean frailty scores (*p* = 0.006). Although the study reported a significant improvement in functional independence (*p* < 0.001), this was assessed to be very low-certainty evidence (Appendix 5). Due to the lack of a control group, it is not possible to conclude whether the results of this study are attributable to the CGA or the TAVI.

The second included study was a quasi-experimental cohort study [22]. A CGA was conducted a day prior to the TAVI procedure by a geriatrician. The findings of the CGA and corresponding recommendations were conveyed to the “heart surgeons” via a standardised paper report form. The CGA covered “physical function and mobility, basic ADL and instrumental ADL, cognitive screening, risk assessment of post-interventional delirium, eye and ear function, nutritional screening, frailty screening, depression screening, polypharmacy screening, assessment of comorbidity burden and assessment of quality of life by validated screening tools”.

Within the quasi-experimental design, the intervention group consisted of those undergoing CGA as per the standard care pathway, and then the comparison group consisted of those who did not receive a CGA for “logistical reasons” such as the absence of the geriatrician, lack of time or lost patient registration.

The study demonstrated that there was no significant improvement in post-operative delirium, LoS, or functional independence in those undergoing CGA. However, the authors acknowledge that performing a CGA the day before the TAVI may not give adequate time for the resulting recommendations to be implemented. There was no data regarding whether the recommendations were acted upon by the surgical team.

#### (ii) Multi-component intervention

There were seven studies analysing the impact of interventions with multiple components[23–29]. All of these studies were observational in nature. Five were conducted within an inpatient setting [23–27, 29] and included an exercise programme alongside various combinations of cardiovascular risk reduction; psychological support; medication review; nutritional assessment. Butter et al. [27] also compared “geriatric rehabilitation”, with “cardiac rehabilitation”, and a control. Those with a higher degree of dependency, defined as a Barthel score of under 70, were selected for geriatric rehabilitation which consisted of a “pre-set treatment plan” delivering “holistic care and consideration of somatic and psychologic[al], and social” elements. They reported 6-month mortality significantly reduced only with cardiac rehabilitation and not with geriatric rehabilitation, as might be expected given the geriatric rehabilitation group were preselected for their lower functional ability at baseline. Overall, these observational studies suggested QoL significantly improved [23, 25, 29] as did physical performance [23–26, 29]. Results were mixed in regards to improvements in functional independence [23, 25, 26, 29] and improvements in mental health [23–25, 29].

The remaining study, by Imran et al. [28] was an observational study of an outpatient post-TAVI multi-component intervention. The intervention consisted of structured, individualised exercise sessions, and advice regarding nutrition, stress and mood management. They reported that the mental composite score, but not the physical composite score, from the Short-Form 36 improved. The authors also describe a significant improvement in physical performance and mental health, but no significant impact on functional independence or nutritional status.

#### (iii) Single-component intervention

##### (a) Exercise-based interventions

Overall, 14 studies assessed 13 different exercise-based interventions. Two of these studies [30, 31] were observational studies which were conducted for an unspecified duration whilst the patient was hospitalised [30, 31]. Both study interventions included at least twice daily exercise sessions on 5 or more days of the week. These studies either had no control group or used patients undergoing surgical aortic valve replacement (sAVR) as a comparator group. Both studies reported a significantly improved level of functional independence and physical performance from baseline to follow-up but no significant impact on levels of anxiety or depression.

Two observational studies [37, 43] looked at the effect of early mobilisation post-procedure compared with standard care. The comparison groups in both studies were patients who had undergone TAVI prior to the intervention being implemented. Both studies found a significantly reduced LoS [37, 43], however, there was no significant difference in-hospital or 30-day mortality [43].

There was one multi-centred, clustered, RCT comparing early mobilisation post-procedure with standard care [42]. The intervention also included guidelines for “quality of care”. This consisted of measures such as providing patient and family education on potential complications, echo-guided or angio-guided access, correct anticoagulation prescribing, suspending nephrotoxic medications, and hydrating the patient before and after the procedure. 1829 participants were enrolled across 20 centres. The authors reported that the intervention significantly reduced LoS and increased the likelihood of being discharged within 3 days of the procedure. There was no significant change in 30-day mortality and 30-day rehospitalisation for cardiac causes.

One paper [39] describes an observational feasibility study of individualised web-based exercise training delivered in the participant’s home. The programme of exercise started 1-week post-TAVI and had a duration of 12 weeks, with first 8 weeks being supervised by physiotherapists. A total of 15 participants were enrolled, with 7 participants completing the study. The results showed a significant improvement in the 6-min walk test (6MWT) and handgrip strength, but a non-significant change in the gait speed, 30-s sit-to-stand test, and QoL. In terms of feasibility, they noted a low recruitment and retention rate. Lack of access to the internet within the participant’s home or poor data coverage were the most common cause for exclusion from participation.

Five papers [32–34, 36, 41] reported the results of three randomised controlled feasibility/pilot studies focused on outpatient exercise-based interventions. Pressler et al. [32, 33] compared participation in an 8-week long programme of 2–3 times weekly exercise sessions with a standard-care control group. They included patients who had undergone a TAVI within the previous 6 months. Rogers et al. [34] compared a 6-week programme of once weekly exercise sessions initiated 1-month post-TAVI with a standard-care control group. Lindman et al. [36] measured the effects of combining an iPad with activity monitoring, personalised daily step goals, and daily resistance exercises for 6-weeks post-procedure. Vitez et al. [41] evaluated 8–12 weeks of supervised outpatient exercise training compared with unsupervised, regular exercise. Noting that they are pilot studies and without power calculations, they found no improvement in their functional independence, physical performance, frailty score, or anxiety or depression [34, 36, 41]. Initial mixed improvements in QoL scores were not sustained at 24 months [32, 33, 36, 41]. There were variable improvements in peak oxygen uptake [32, 33, 41].

Weber et al. [38] reported on an RCT measuring the effect of a combined outpatient pre-TAVI and inpatient post-TAVI exercise-based intervention. The control group received an inpatient post-TAVI intervention of lesser intensity. The primary endpoint, defined as 35% reduction in rehospitalisation or mortality at 90 days, was not met, however, the intervention significantly reduced incidence of pneumonia and LoS. This study was assessed to be at moderate risk of bias due to a deviation from the stated intervention protocol and because not all outcomes were reported. The trial was also significantly underpowered, having only recruited 108 of the 220 participants required. The authors report the under recruitment was due to difficulty identifying participants who were able to complete the minimum 2-week programme of pre-TAVI exercise.

Hu et al. [40] conducted an RCT comparing outpatient “moderate-intensity continuous training (MICT)” with standard care. The MICT was commenced at least 1 month after the TAVI and consisted of three 45-min sessions every week for 3 months. The authors demonstrated a significant improvement in the peak VO_2_ and 6MWT but a non-significant change in QoL.

##### (b) CBT-based intervention

Edwards et al. [35] analysed the effect of CBT post-TAVI. They conducted an RCT comparing four 30–60 min bedside based sessions of CBT whilst the patient was an inpatient, with a standard-care control group. The results showed no statistically significant difference between the control and intervention group in depression or anxiety symptoms, or in quality of life at 1-month post-procedure. This study was also found to be at high risk of bias due to deviations from the intervention protocol, a significant volume of missing data due to loss to follow-up, and an inappropriate choice of outcome measures.

**Table 1:** Characteristics of the included studies

Study	Study size	Participant demographics Mean age ± SD (years) % male	Study design	Intervention (n =)	Comparator (n =)	Outcome domains Measured (tool used) *Statistically significant positive effect of the intervention with respect to this outcome measure **Statistically significant negative effect of the intervention with respect to this outcome measure	Involvement of professional with geriatrics training?	Assessment of frailty? (Measure used, proportion of intervention population)
*CGA*								
Yu, et al. 2021, China [21]	90	74.7 ± 8.1 60% male	Observational, prospective	CGA (90)	Single arm study, compared before and after TAVI and intervention (0)	Functional Independence (Barthel Index*) Physical Performance (6MWD*, MET*) Nutritional Status (MNA*) Cognition (MMSE*) Mental Health (HADS-A**, HADS-D)	No	Yes (fried frailty scale, 83%)
Schwesinger, et al. 2024, Switzerland [22]	435	81.0 ± 5.6 56.4% male	Quasi-experimental cohort study	CGA (254)	No CGA (181)	Cognition (PoD) LoS Functional independence (change in SPI)	Yes	Yes (however, missing data from all of the comparator group and some of intervention group. So proportion not state here)
*Multi-component intervention*								
Zanettini, et al. 2014, Italy [23]	60	83.5 ± 5.0 46.7% male	Observational, prospective	Inpatient Multicomponent intervention (60)	Single arm study, compared before and after TAVI and intervention (0)	QoL (EQ VAS*) Functional independence (Barthel Index*) Physical Performance (6MWT*)	No	Not formally assessed/reported
Völler, et al. 2015, Germany [24]	76	80.30 ± 6.15 42.1% male	Observational, retrospective	Inpatient Multicomponent intervention (76)	Single arm study, compared before and after TAVI and intervention (0)	Physical performance (6MWT*, exercise capacity*) Mental health (HADS)	No	Not formally assessed/reported
Eichler, et al. 2017, Germany [25]	136	80.6 ± 5.0 47.8% male	Observational, prospective	Inpatient Multicomponent intervention (136)	Single arm study, compared before and after TAVI and intervention (0)	QoL (SF-12 PCS*, SF-12 MCS*) Functional independence (ADL, iADL) Physical performance (6MWD*, exercise capacity*, proportion with mobility disability**, TUG**) Cognition (MMSE*) Mental health (HADS-A**, HADS-D)	No	Yes (composite frailty index calculated from multiple measures, 36.9%)
Genta, et al. 2017, Italy [26]	65	80 ± 5 37% male	Observational, prospective	Inpatient multicomponent intervention (65)	Single arm study, compared before and after TAVI and intervention (0)	Functional Independence (Barthel Index*) Physical Performance (6MWT*) Other (Morse Fall Scale*)	No	Not formally assessed/reported
Butter, et al. 2018, Germany, [27]	1017	80.7 ± 6.0 44.5% male	Observational, Prospective	Inpatient multicomponent intervention - Cardiac rehabilitation (n = 435), Geriatric Rehabilitation (216)	Standard care (366)	All-cause mortality** Hospitalisation rate	Not stated explicitly	Not formally assessed/reported
Imran, et al. 2018, USA [28]	24	70.9 ± 1.0 65.1% male	Observational, retrospective	Outpatient multicomponent intervention (24)	Single arm study, compared before and after TAVI and intervention (0)	QoL (SF-36 PCS, SF-36 MCS*) Functional independence (ENRICHD Social Support Survey) Physical Performance (Exercise Duration*, Exercise Intensity*) Nutritional Status (RYP Food Survey) Mental Health (GAD-7, PHQ-9**, PANAS*)	No	Not formally assessed/reported
Kleczynski, et al. 2021, Poland [29]	105	80 ± 4.5 40% male	Observational, retrospective	Inpatient multicomponent intervention (52)	Standard care (53)	QoL (KCCQ at 30 days and 6 months*, KCCQ at 12 months) Functional independence (KI ADL at 30 days and 6 months*, KI ADL at 12 months) Physical Performance (6MWT*, 5MWT**, HGS*) Mental Health (HADS-A, HADS-D)	No	Not formally assessed/reported
*Single-component intervention*								
Fauchere, et al. 2014, Switzerland [30]	34	79 ± 6.0 40% male	Observational, retrospective	Inpatient exercise-based intervention (34)	Single arm study, compared before and after TAVI and intervention (0)	Functional independence (FIM*) Physical performance (6MWT*) Mental health (HADS)	No	Not formally assessed/reported
Russo, et al. 2014, Italy [31]	138	82.1 ± 3.6 40% male	Observational, Prospective	Inpatient Exercise-based intervention (78)	Surgical aortic valve replacement (sAVR) (80)	Functional Independence (Barthel index) Physical performance (6MWT, CPET)	No	Not formally assessed/reported
Pressler, et al. 2016, Germany [32]	30	81 ± 6 55.6% male	RCT (pilot study)	Outpatient exercise-based intervention (15)	Standard care (15)	QoL (KCCQ*, SF12) Physical performance (VO_2_ peak * VO2AT*, 6MWT)	No	Not formally assessed/reported
Pressler, et al. 2018, Germany [33]	17	82 ± 7 53% male	This paper presented 2 year follow-up data from Pressler et al. 2016 [29]	Outpatient Exercise-based intervention (10)	Standard care (7)	QoL (KCCQ, SF12) Physical Performance (VO_2_ peak, VO2AT*, 6MWT)	No	Not formally assessed/reported
Rogers, et al. 2018, UK [34]	27	82.0 ± 4.8 44.4% male	RCT (pilot study)	Outpatient exercise-based intervention (13)	Standard care (14)	Functional independence (Nottingham EADL) Physical performance (6MWT) Mental health (HADS-A, HADS-D)	No	Yes (Fried frailty score, 20.0%; Edmonton Frailty score, no proportions given however mean score reported as 5.17)
Edwards, et al. 2020, USA [35]	146	82.3 ± 7.5 in standard care arm 82.5 ± 9.3 in intervention arm 56.8% male	RCT	Cognitive based therapy (80)	Standard care (66)	QoL (SF-12) Mental health (BDI-II, STAI-YI, MLHFQ)	No	Yes (5MWT, 34%)
Lindman, et al. 2021, USA [36]	50	76 ± 9 in standard care arm 76 ± 7 in intervention arm 66% male	RCT (pilot study)	Outpatient exercise-based intervention (25)	Standard care (25)	QoL (KCCQ) Physical performance (SPPB, Gait Speed, STS Time, Balance Score, 6MWT, HGS, daily active minutes*, daily active minutes of moderate to high intensity*) Mental health (PROMIS10 global mental health, PROMIS10 CAT depression score)	No	Yes (slow gait speed, 18%; SPPB < 10, 40%)
Myszenski, et al. 2021, USA [37]	189	79.5 ± 11.2 in standard care arm 81.6 ± 8.4 in intervention arm 58.2% male	Observational, retrospective	Early mobilisation intervention (115)	Standard care (74)	LoS** Proportion discharged home	No	Yes (5MWT > 6 s, 60%)
Weber, et al. 2021, Germany [38]	108	82.0 ± 5.5 49.1% male	RCT	Inpatient and outpatient exercise-based intervention (58)	Standard care (n = 50)	90-day mortality 90-day rehospitalisation rates Incidence of Pneumonia** LoS**	No	Not formally assessed/reported
Brocki et al. 2023, Denmark [39]	15	Median 84 years (IQR 82–87) 40% male	Observational, prospective, feasibility study	Home web-based exercise training (15)	Single arm study, compared before and after TAVI and intervention (0)	QoL (HeartQoL, EQ VAS) Physical performance (6MWT*, HGS*, gait speed, 30STS)	No	Yes (Tilburg Frailty Indicator, 33%; gait speed < 7 m/s, 20%)
Hu et al. 2023, China [40]	66	70.6 ± 6.6 54.5% male	RCT	Outpatient exercise-based intervention (33)	Standard care (33)	QoL (SF12) Physical performance (VO_2_ peak*, 6MWT*)	No	Not formally assessed/reported
Vitez et al. 2023, Slovenia [41]	23	81.1 ± 1 39% male	RCT (pilot study)	Outpatient exercise-based intervention (10)	Unsupervised home-based exercise (13)	QoL (EQ-5D-5L, SF-36) Physical performance (VO_2_ peak, exercise time, 6MWT, HGS)	No	Not formally assessed/reported
Durand et al. 2024, France [42]	1829	81.9 ± 6.6 55% male	RCT	Early mobilisation intervention (969)	Standard care (860)	30-day mortality 30-day rehospitalisation for cardiovascular events LoS** Discharge within 3 days of procedure*	No	Yes (however, unclear what frailty measure was used so proportion not stated here)
Frank et al. 2024, Europe [43]	2388	79.9 ± 6.8 in standard care arm 79.8 ± 6.6 in intervention arm	Observational, prospective	Early mobilisation intervention (1491)	Standard care (897)	In-hospital mortality 30-day mortality LoS**	No	Yes (however, unclear what frailty measure was used so proportion not stated here)

*ADL* Activities of Daily Living, *CPET* cardiopulmonary exercise testing, *EQ VAS* EuroQol Visual Analogue Scale, *FIM* Functional Independence measure, *GDS* Geriatric Depression Scale, *HADS* Hospital Anxiety and Depression Scale, *HGS* Handgrip strength, *HeartQoL* health-related quality of life questionnaire, *IADL* Instrumental Activities of Daily Living, *KCCQ* Kansas City Cardiomyopathy Questionnaire, *KI ADL* Katz index of Independence of Activities in Daily Living, *LoS* length of stay, *METs* Metabolic Equivalent, *MMSE* mini-mental state examination, *MNA* mini nutritional assessment, *PANAS* Positive and Negative Affect Schedule, *PHQ-9* Patient Health Questionnaire – 9, *PoD* Postoperative delirium, *SF-36* Short Form 36, *SF-12 MCS* Short Form 12 mental component score, *SF-12 PCS* Short Form 12 physical component score, *SPI* self-care index, *TUG* Timed Up and Go, *30STS* 30-s sit-to-stand test, *5MWT* 5 m walk time, *6MWD* 6 min walk distance

## Discussion

Despite considerable evidence that frailty is directly related to poor outcomes post-TAVI, this systematic review found an absence of evidence to support the use of CGA, or interventions targeting the domains of CGA, as a treatment to improve outcomes for this patient group. In expanding our search to include studies of components of CGA, we had aimed to develop an evidence base informing interventions that address issues identified during a CGA assessment. However, we found only observational studies, pilot RCTs, and RCTs that had at least a moderate risk of bias, all of which were insufficient to inform a tailored CGA approach.

No studies specifically targeted those living with frailty, the population with the most to gain from a CGA approach given the known impact of frailty on TAVI outcomes [8], paired with CGA being the gold standard for the management of frailty in older people [8, 49]. Only 10 of the included studies measured frailty as a baseline characteristic [21, 22, 25, 34–37, 39, 42, 43]. When frailty was measured the assessment scale used was varied, despite the EFT having been shown to be the best predictor for death and disability in adults undergoing aortic valve replacement [8]. Using the EFT to define the study population would enable interventions to be trialled on those most likely to benefit.

Although our review has identified two studies of CGA prior to TAVI, there has been work reviewing the use of either CGA or multi-component interventions in other groups of patients with CVD. A geriatric nurse-led CGA in a non-randomised study involving inpatient cardiovascular patients aged 75 or over demonstrated a statistically significant, but not clinically significant, difference in functional status between the control and intervention group [15]. Recently, the TARGET-EFT trial reported that a multi-component intervention for inpatient older adults with frailty and CVD, including those with symptomatic valvular disease, led to improvements in health-related QoL and mental well-being, but had no impact on disability levels [50]. We also know from previous studies that geriatrician-led CGA in the perioperative setting can improve postoperative outcomes including LoS and medical complications, and is cost effective [51]. This work in CVD and peri-operative care suggests good face validity for the use of CGA prior to TAVI.

Future studies would benefit from applying the principles described by the Medical Research Council guidelines for research into complex interventions, such as CGA [52]. This guidance acknowledges the difficulties in studying an intervention which has multiple interacting components, requires behavioural changes in those delivering and receiving the intervention, and has multiple groups involved in the intervention, and offers suggestions on how to account for these complexities within the study design. To improve the quality of forthcoming trials, these guidelines need to be incorporated from the outset of study design alongside a cost-effectiveness analysis. Future studies would also benefit from ensuring that they measure the key outcomes identified as being important to older people, in order to promote a better quality of care for older adults and to facilitate comparisons between studies [53]. Ideally, upcoming studies should take the form of an adequately powered RCT to ensure a strong evidence base for developing clinical guidelines.

Any future work should also be pragmatic and suitable for both the target population and the healthcare system in which the intervention would be applied. Exercise programmes were a key intervention in many of the studies. However, some required prolonged inpatient stays or frequent outpatient exercise classes. Interventions such as these may exclude portions of the target population as they are not able to engage with the service. For instance, there may be difficulties with travelling to such classes. It may also be impractical and unaffordable in most healthcare systems, and contrary to generally accepted best practice of avoiding prolonged admissions for those living with frailty. Indeed, this factor led the certainty of evidence to be downgraded as the study interventions would not be easily replicable within wider health services [20].

Thirteen of the 20 studies included in this review involved evaluating a form of exercise programme in individuals undergoing TAVI. The studies resulted in very low or low certainty evidence and were assessed to be at moderate or serious risk of bias. When conducting new studies, we need to be sure of equipoise and that we are not repeating work that has already been conducted. If, in the case where research questions are being addressed again, this should be with the purpose of improving on the quality of previous studies. On rerunning the database search for this review, we noted a protocol for an RCT which is currently in progress and seeks to evaluate an exercise intervention in people undergoing TAVI [54]. It will be important that this work builds on previous studies. Of note, all studies were assessed to be at moderate or serious risk of confounding. Often the study design did not appropriately control for the significant confounder of undergoing a TAVI procedure and the expected improvement in health following this. Addressing this issue should be a focus of future work.

The need for well-conducted studies in older adults undergoing TAVI is compounded by the increasing interest in the role of geriatricians, geriatric principles, and frailty in CVD and cardiovascular interventions [55–57]. The European Society of Cardiology has established a Task Force on Geriatric Cardiology with a focus on, “frailty in cardiovascular disease” [57]. The European Union Geriatric Medicine Society (EUGMS) also released a position statement which stated “geriatricians should routinely perform CGA in patients with severe aortic stenosis scheduled to undergo sAVR or TAVI and during long-term follow-up” [58]. Although, interestingly, EUGMS have emphasised the role of CGA as a predictive tool for outcomes post-procedure, rather than the use of CGA as an intervention to improve outcomes [59].

This review has demonstrated there is minimal evidence on which to base the recommendations for applying the expertise of a geriatrician and a lack of cost-effectiveness data to support the call for their input. Thus, the role of geriatrics within this population needs to be better defined and evidenced before changes to clinical practice are implemented. This is particularly important given the shortage of geriatricians to fill such roles [60] and the evidence suggesting non-geriatrician led CGA is challenging to implement [61]. The recent HoW-CGA study attempted to implement the delivery of CGA by non-geriatricians within the perioperative setting, but were unsuccessful [62]. In the subsequent discussions of why the trial was not able to change practice, it was suggested that CGA needs to be geriatrician-led for it to be effective [61, 63].

There are limitations to this review including the paucity of data to draw conclusions and the lack of meta-analysis. Due to the heterogeneity of outcome measures and methodological weaknesses in the included studies, the authors were not able to conduct a meta-analysis.

## Conclusion

There is a lack of evidence to determine whether CGA, or related interventions, improve outcomes for older adults post-TAVI. The strong evidence base for perioperative CGA, alongside the results of this review, support the need for well-designed trials to evaluate whether CGA improves outcomes for older adults with frailty who are undergoing TAVI and therefore inform potential implementation in TAVI pathways.

## Supplementary Information

Below is the link to the electronic supplementary material.

Supplementary file1 (DOCX 31 kb)

Supplementary file2 (DOCX 20 kb)

Supplementary file3 (DOCX 27 kb)

Supplementary file4 (DOCX 47 kb)

Supplementary file5 (DOCX 17 kb)

## Data Availability

Data sharing is not applicable to this article as no datasets were generated or analysed during the current study. The individual articles included in this review are available from the publishing journal.
