# Supplementary material for: Comprehensive geriatric assessment, and related interventions, to improve outcomes for older patients undergoing transcatheter aortic valve implantation (TAVI): a systematic review
Source: Eur Geriatr Med. 2024 Sep 27;15(6):1615–30. doi: 10.1007/s41999-024-01035-5 (PMC11631815; doi:10.1007/s41999-024-01035-5)
Supplement: Supplementary file 2 — Supplementary file2 (DOCX 20 kb) [file 41999_2024_1035_MOESM2_ESM.docx]

Appendix 2 – search strategy

**MEDLINE(R) (OVID Platform)**

1 exp AGED/

2 exp GERIATRICS/

3 exp FRAILTY/

4 exp GERIATRICIANS/

5 exp MULTIMORBIDITY/

6 Aged.mp.

7 Ag?ing.mp.

8 Elderl*.mp.

9 Senior.mp.

10 (Old* adj (People or Person* or resident* or adult* or patient*)).mp.

11 Geriatr*10.mp.

12 Gerontolog*10.mp.

13 Frail*.mp.

14 ("multimorbidity" or "multi-morbidity" or "multiple comorbidit*" or "multiple chronic conditions").mp.

15 or/1-14

16 exp TRANSCATHETER AORTIC VALVE IMPLANTATION/

17 TAVI.mp.

18 TAVR.mp.

19 Transcatheter Aortic Valve Replacement.mp.

20 Trans-catheter Aortic Valve Replacement.mp.

21 Transcatheter Aortic Valve Implantation.mp.

22 Trans-catheter Aortic Valve Implantation.mp.

23 ((Percutan* or Transcath* or Transapical or transventricular or transfemoral or transarterial) adj3 aortic valve).mp.

24 ((Percutan* or Trans-cath* or Trans-apical or trans-ventricular or trans-femoral or trans-arterial) adj3 aortic valve).mp.

25 or/16-24

26 exp GERIATRIC ASSESSMENT/

27 exp HEALTH SERVICES FOR THE AGED/

28 exp Needs Assessment/

29 exp PREVENTIVE MEDICINE/

30 exp PREOPERATIVE CARE/

31 exp Patient Care Team/

32 CGA.mp.

33 Comprehensive Geriatric Assessment.mp.

34 Geriatric Assessment.mp.

35 Comprehensive Assessment.mp.

36 (Geriatric adj3 (assess* or evaluat* or apprais* or function or functioning or comprehensive or patient care team or consultation or patient* education or interprofession* or inter-profession* or interdisciplin* or inter-disciplin* or multi-disciplin* or multidisciplin* or rehab*)).mp.

37 (Elderly adj3 (assess* or evaluat* or apprais* or function or functioning or comprehensive or patient care team or consultation or patient* education or interprofession* or inter-profession* or interdisciplin* or inter-disciplin* or multi-disciplin* or multidisciplin* or rehab*)).mp.

38 Prehabilitation.mp.

39 Geriatric Rehabilitation.mp.

40 Multimodal Intervention.mp.

41 Multi-modal intervention.mp.

42 Multicomponent intervention.mp.

43 multi-component intervention.mp.

44 (Charlson Comorbidity Index or Physical Self-Maintenance Scale or Barthel Index or Clinical Frailty Scale or Fried Frailty Score or Lawton Index or Katz ADL or Essential Frailty Toolset or EFT).mp.

45 (Grip Strength or SPPB or Short Physical Performance Battery or TUGT or "Timed Up and Go test" or "Six Minute Walk* Test" or "6 Minute Walk Test" or Gait Assessment or Balance Assessment).mp.

46 (Rehabilitation or Exercise Program* or Physiotherap* or Exercises or Prehab).mp.

47 (Geriatric Depression Sc* or PHQ* or Patient Health Questionnaire or MOCA or Montreal Cognitive Assessment or MMSE or Mini Mental State Examination or Mini-Cog).mp.

48 (MUST or BMI or Mini Nutritional Assessment or MNA or Diet* or Nutri*).mp.

49 (Medication Review or Medications Rationalisation or Pharmac*).mp.

50 or/26-49

51 15 and 25 and 50

52 exp clinical study/

53 exp clinical trial/

54 exp cohort analysis/

55 exp prospective study/

56 exp longitudinal study/

57 exp case control study/

58 clinical trial.pt.

59 randomized controlled trial.pt.

60 observational study.pt.

61 clinical study.pt.

62 clinical trial protocol.pt.

63 controlled clinical trial.pt.

64 pragmatic clinical trial.pt.

65 journal article.pt.

66 (random* or placebo* or single blind* or double blind* or triple blind*).ti,ab.

67 (trial or multicenter or multi center or multi-center or multicentre or multi centre or multi-centre).ti.

68 (intervention? or effect? or impact? or controlled or control group? or (before adj5 after) or (pre adj5 post) or ((pretest or pre test) and (posttest or post test)) or quasiexperiment* or quasi experiment* or quasi-experiment* or pseudo experiment* or pseudoexperiment* or pseudo-experiment* or evaluat* or time series or time point? or repeated measur*).ti,ab.

69 (case* and control*).ti,ab.

70 cohort*.ti,ab.

71 group*.ab.

72 or/52-71

73 meta-analysis.pt.

74 systematic review.pt.

75 editorial.pt.

76 case reports.pt.

77 letter.pt.

78 literature review.ti.

79 or/73-78

80 72 not 79

81 51 and 80

82 limit 81 to yr="1980 -Current"

**EMBASE (OVID Platform)**

1 exp AGED/

2 exp GERIATRICS/

3 exp FRAILTY/

4 exp GERIATRICIANS/

5 exp GERIATRIC DISORDER/

6 exp MULTIMORBIDITY/

7 Aged.mp.

8 Ag?ing.mp.

9 Elderl*.mp.

10 Senior.mp.

11 (Old* adj (People or Person* or resident* or adult* or patient*)).mp.

12 Geriatr*10.mp.

13 Gerontolog*10.mp.

14 Frail*.mp.

15 ("multimorbidity" or "multi-morbidity" or "multiple comorbidit*" or "multiple chronic conditions").mp.

16 or/1-15

17 exp TRANSCATHETER AORTIC VALVE IMPLANTATION/

18 TAVI.mp.

19 TAVR.mp.

20 Transcatheter Aortic Valve Replacement.mp.

21 Trans-catheter Aortic Valve Replacement.mp.

22 Transcatheter Aortic Valve Implantation.mp.

23 Trans-catheter Aortic Valve Implantation.mp.

24 ((Percutan* or Transcath* or Transapical or transventricular or transfemoral or transarterial) adj3 aortic valve).mp.

25 ((Percutan* or Trans-cath* or Trans-apical or trans-ventricular or trans-femoral or trans-arterial) adj3 aortic valve).mp.

26 or/17-25

27 exp GERIATRIC ASSESSMENT/

28 exp HEALTH SERVICES FOR THE AGED/

29 exp Needs Assessment/

30 exp PREVENTIVE MEDICINE/

31 exp PREOPERATIVE PERIOD/

32 exp Patient Care Team/

33 exp GERIATRIC REHABILITATION/

34 CGA.mp.

35 Comprehensive Geriatric Assessment.mp.

36 Geriatric Assessment.mp.

37 Comprehensive Assessment.mp.

38 (Geriatric adj3 (assess* or evaluat* or apprais* or function or functioning or comprehensive or patient care team or consultation or patient* education or interprofession* or inter-profession* or interdisciplin* or inter-disciplin* or multi-disciplin* or multidisciplin* or rehab*)).mp.

39 (Elderly adj3 (assess* or evaluat* or apprais* or function or functioning or comprehensive or patient care team or consultation or patient* education or interprofession* or inter-profession* or interdisciplin* or inter-disciplin* or multi-disciplin* or multidisciplin* or rehab*)).mp.

40 Prehabilitation.mp.

41 Geriatric Rehabilitation.mp.

42 Multimodal Intervention.mp.

43 Multi-modal intervention.mp.

44 Multicomponent intervention.mp.

45 multi-component intervention.mp.

46 (Charlson Comorbidity Index or Physical Self-Maintenance Scale or Barthel Index or Clinical Frailty Scale or Fried Frailty Score or Lawton Index or Katz ADL or Essential Frailty Toolset or EFT).mp.

47 (Grip Strength or SPPB or Short Physical Performance Battery or TUGT or "Timed Up and Go test" or "Six Minute Walk* Test" or "6 Minute Walk Test" or Gait Assessment or Balance Assessment).mp.

48 (Rehabilitation or Exercise Program* or Physiotherap* or Exercises or Prehab).mp.

49 (Geriatric Depression Sc* or PHQ* or Patient Health Questionnaire or MOCA or Montreal Cognitive Assessment or MMSE or Mini Mental State Examination or Mini-Cog).mp.

50 (MUST or BMI or Mini Nutritional Assessment or MNA or Diet* or Nutri*).mp.

51 (Medication Review or Medications Rationalisation or Pharmac*).mp.

52 or/27-51

53 16 and 26 and 52

54 exp clinical trial/

55 exp controlled study/

56 exp cohort analysis/

57 exp prospective study/

58 exp longitudinal study/

59 exp follow up/

60 exp case control study/

61 (random* or placebo* or single blind* or double blind* or triple blind*).ti,ab.

62 (trial or multicenter or multi center or multi-center or multicentre or multi centre or multi-centre).ti.

63 group*.ab.

64 (intervention? or effect? or impact? or controlled or control group? or (before adj5 after) or (pre adj5 post) or ((pretest or pre test) and (posttest or post test)) or quasiexperiment* or quasi experiment* or quasi-experiment* or pseudo experiment* or pseudoexperiment* or pseudo-experiment* or evaluat* or time series or time point? or repeated measur*).ti,ab.

65 cohort*.ti,ab.

66 (case* and control*).ti,ab.

67 or/54-66

68 editorial.pt.

69 letter.pt.

70 review.pt.

71 systematic review.ti.

72 literature review.ti.

73 meta analysis.ti.

74 or/68-73

75 67 not 74

76 53 and 75

77 limit 76 to yr="1980 -Current"

**CINAHL (EBSCO Platform)**

S1 (MH "Aged+")

S2 (MH "Geriatrics")

S3 (MH "Frailty Syndrome")

S4 (MH "Geriatricians")

S5 TX Aged

S6 TX Ag#ing

S7 TX Elderl*

S8 TX Senior

S9 TX Old* W3 (People or Person* or resident* or adult* or patient*)

S10 TX Geriatr*

S11 TX Gerontol*

S12 TX Frail*

S13 TX "multimorbidity" OR "multi-morbidity" OR "multiple comorbidit*" OR "multiple chronic conditions"

S14 S1 OR S2 OR S3 OR S4 OR S5 OR S6 OR S7 OR S8 OR S9 OR S10 OR S11 OR S12 OR S13

S15 (MH "Transcatheter Aortic Valve Implantation")

S16 TX TAVI

S17 TX TAVR

S18 TX transcatheter aortic valve replacement OR trans-catheter aortic valve replacement

S19 TX transcatheter aortic valve implantation OR trans-catheter aortic valve implantation

S20 TX (Percutan* or Transcath* or Transapical or transventricular or transfemoral or transarterial) W4 Aortic valve

S21 TX (Trans-cath* or Trans-apical or trans-ventricular or trans-femoral or trans-arterial) W4 Aortic valve

S22 S15 OR S16 OR S17 OR S18 OR S19 OR S20 OR S21

S23 (MH "Geriatric Assessment+")

S24 (MH "Health Services for the Aged")

S25 (MH "Needs Assessment")

S26 (MH "Preoperative Care+")

S27 (MH "Multidisciplinary Care Team+")

S28 (MH "Rehabilitation, Geriatric")

S29 (MH "Functional Assessment+")

S30 TX CGA

S31 TX comprehensive geriatric assessment

S32 TX Comprehensive Assessment

S33 TX Geriatric Assessment

S34 TX (Geriatric W3 (assess* or evaluat* or apprais* or function or functioning or comprehensive or patient care team or consultation or patient* education or interprofession* or inter-profession* or interdisciplin* or inter-disciplin* or multi-disciplin* or multidisciplin* or rehab*)

S35 TX (Elderly W3 (assess* or evaluat* or apprais* or function or functioning or comprehensive or patient care team or consultation or patient* education or interprofession* or inter-profession* or interdisciplin* or inter-disciplin* or multi-disciplin* or multidisciplin* or rehab*)

S36 TX prehabilitation

S37 TX geriatric rehabilitation

S38 TX multimodal intervention or multi-modal intervention

S39 TX multicomponent intervention or multi-component intervention

S40 TX (Charlson Comorbidity Index or Physical Self-Maintenance Scale or Barthel Index or Clinical Frailty Scale or Fried Frailty Score or Lawton Index or Katz ADL or Essential Frailty Toolset or EFT)

S41 TX (Grip Strength or SPPB or Short Physical Performance Battery or TUGT or "Timed Up and Go test" or "Six Minute Walk* Test" or "6 Minute Walk Test" or Gait Assessment or Balance Assessment)

S42 TX (Rehabilitation or Exercise Program* or Physiotherap* or Exercises or Prehab)

S43 TX (Geriatric Depression Sc* or PHQ* or Patient Health Questionnaire or MOCA or Montreal Cognitive Assessment or MMSE or Mini Mental State Examination or Mini-Cog)

S44 TX (MUST or BMI or Mini Nutritional Assessment or MNA or Diet* or Nutri*)

S45 TX (Medication Review or Medications Rationalisation or Pharmac*)

S46 S23 OR S24 OR S25 OR S26 OR S27 OR S28 OR S29 OR S30 OR S31 OR S32 OR S33 OR S34 OR S35 OR S36 OR S37 OR S38 OR S39 OR S40 OR S41 OR S42 OR S43 OR S44 OR S45

S47 S14 AND S22 AND S46

S48 (MH "Experimental Studies+")

S49 (MH "Prospective Studies+")

S50 (MH "Quasi-Experimental Studies+")

S51 AB (random* or placebo* or single blind* or double blind* or triple blind*)

S52 AB (trial or multicenter or multi center or multi-center or multicentre or multi centre or multi-centre)

S53 AB group*

S54 AB (intervention? or effect? or impact? or controlled or control group? or (before adj5 after) or (pre adj5 post) or ((pretest or pre test) and (posttest or post test)) or quasiexperiment* or quasi experiment* or quasi-experiment* or pseudo experiment* or pseudoexperiment* or pseudo-experiment* or evaluat* or time series or time point? or repeated measur*)

S55 AB cohort*

S56 AB (case* and control*)

S57 S48 OR S49 OR S50 OR S51 OR S52 OR S53 OR S54 OR S55 OR S56

S58 PT editorial

S59 PT letter

S60 PT review

S61 PT systematic review

S62 PT meta analysis

S63 S58 OR S59 OR S60 OR S61 OR S62

S64 S57 NOT S63

S65 S47 and S64 Limiters - Published Date: 19800101-20221231

**Cochrane Central Register of Controlled Trials (CENTRAL)**

#1 MeSH descriptor: [Aged] explode all trees

#2 MeSH descriptor: [Geriatrics] explode all trees

#3 MeSH descriptor: [Frailty] explode all trees

#4 MeSH descriptor: [Geriatricians] explode all trees

#5 MeSH descriptor: [Multimorbidity] explode all trees

#6 ("aged"):ti,ab,kw

#7 (Ag*ng):ti,ab,kw

#8 (elderl*):ti,ab,kw

#9 (Senior):ti,ab,kw

#10 (Old* near (people OR person* OR resident* OR adult* OR patient*)):ti,ab,kw

#11 (Geriatr*):ti,ab,kw

#12 (Gerontolog*):ti,ab,kw

#13 (Frail*):ti,ab,kw

#14 (multimorbid* or multi-morbid*):ti,ab,kw

#15 {OR #1-#14}

#16 MeSH descriptor: [Transcatheter Aortic Valve Replacement] explode all trees

#17 (TAVI):ti,ab,kw

#18 (TAVR):ti,ab,kw

#19 (Transcatheter Aortic Valve Replacement):ti,ab,kw

#20 (Trans-catheter Aortic Valve Replacement):ti,ab,kw

#21 (Transcatheter Aortic Valve Implantation):ti,ab,kw

#22 (Trans-catheter Aortic Valve Implantation):ti,ab,kw

#23 ((Percutan* or Transcath* or Transapical or transventricular or transfemoral or transarterial) NEAR "aortic valve"):ti,ab,kw

#24 ((Trans-cath* or Trans-apical or trans-ventricular or trans-femoral or trans-arterial) NEAR aortic valve):ti,ab,kw

#25 {OR #16-#24}

#26 MeSH descriptor: [Geriatric Assessment] explode all trees

#27 MeSH descriptor: [Health Services for the Aged] explode all trees

#28 MeSH descriptor: [Needs Assessment] explode all trees

#29 MeSH descriptor: [Preventive Medicine] explode all trees

#30 MeSH descriptor: [Preoperative Care] explode all trees

#31 MeSH descriptor: [Patient Care Team] explode all trees

#32 (CGA):ti,ab,kw

#33 (Comprehensive Geriatric Assessment):ti,ab,kw

#34 (Geriatric Assessment):ti,ab,kw

#35 (Comprehensive Assessment):ti,ab,kw

#36 (Geriatric NEAR (assess* or evaluat* or apprais* or function* or comprehensive or "patient care team" or consultation or patient* education or interprofession* or inter-profession* or interdisciplin* or inter-disciplin* or multi-disciplin* or multidisciplin* or rehab*)):ti,ab,kw

#37 ((Elderly NEAR (assess* or evaluat* or apprais* or function* or comprehensive or "patient care team" or consultation or patient* education or interprofession* or inter-profession* or interdisciplin* or inter-disciplin* or multi-disciplin* or multidisciplin* or rehab*))):ti,ab,kw

#38 ("prehabilitation"):ti,ab,kw

#39 (Geriatric Rehabilitation):ti,ab,kw

#40 (Multimodal intervention):ti,ab,kw

#41 (Multi-modal intervention):ti,ab,kw

#42 (Multicomponent intervention):ti,ab,kw

#43 (multi-component intervention):ti,ab,kw

#44 ((Charlson Comorbidity Index or Physical Self-Maintenance Scale or Barthel Index or Clinical Frailty Scale or Fried Frailty Score or Lawton Index or Katz ADL or Essential Frailty Toolset or EFT)):ti,ab,kw

#45 ((Grip Strength or SPPB or Short Physical Performance Battery or TUGT or "Timed Up and Go test" or "Six Minute Walk* Test" or "6 Minute Walk Test" or Gait Assessment or Balance Assessment)):ti,ab,kw

#46 ((Rehabilitation or Exercise Program* or Physiotherap* or Exercises or Prehab)):ti,ab,kw

#47 ((Geriatric Depression Sc* or PHQ* or Patient Health Questionnaire or MOCA or Montreal Cognitive Assessment or MMSE or Mini Mental State Examination or Mini-Cog)):ti,ab,kw

#48 ((MUST or BMI or Mini Nutritional Assessment or MNA or Diet* or Nutri*)):ti,ab,kw

#49 ((Medication Review or Medications Rationalisation or Pharmac*)):ti,ab,kw

#50 {OR #26-#49}

#51 {AND #15, #25, #50} with Cochrane Library publication date Between Jan 1980 and Dec 2021
