# Supplementary material for: Comprehensive geriatric assessment, and related interventions, to improve outcomes for older patients undergoing transcatheter aortic valve implantation (TAVI): a systematic review
Source: Eur Geriatr Med. 2024 Sep 27;15(6):1615–30. doi: 10.1007/s41999-024-01035-5 (PMC11631815; doi:10.1007/s41999-024-01035-5)
Supplement: Supplementary file 3 — Supplementary file3 (DOCX 27 kb) [file 41999_2024_1035_MOESM3_ESM.docx]

Appendix 3 – Reason for exclusion at full text screening

| Reason for exclusion | Comments | Reference |
| --- | --- | --- |
| Content overlap | Conference abstract presenting initial data later reported in full in Weber et al, 2021 (included study) | Weber M, Weigert A, Klein U, Momcilovic D, Schueler R, Oeztuerk C, Pingel S, Stundl A, Sinning J-M, Hammerstingl C, Werner N, Grube E, Nickenig G, Ghanem A. Benefit of pre-and intensified post-procedural physiotherapy in patients with symptomatic aortic stenosis undergoing transcatheter aortic valve implantation (4P-TAVI) study. European heart journal 2016; 37(null): 943‐. |
| Content overlap | Poster presentation - same patient group discussed in further detail in full included report Eichler et al 2017 | Voller H., Eichler S., Harnath A., et al Multicomponent cardiac rehabilitation in patients after transcatheter aortic valve implantation (TAVI)-course of functioning and quality of life. *Eur. Heart J.* 2016;37(Supplement 1):542. doi:10.1093/eurheartj/ehw432 |
| Content overlap | Results provided in more detail in included paper (Pressler et al 2016) | Pressler A, Lechner B, Christle J, Bleiziffer S, Mehilli J, Jochheim D, Hettich I, Halle M. Safety, applicability and outcome of regular exercise training after transcatheter aortic valve implantation: a randomized pilot trial. European journal of preventive cardiology 2015; 22(1 SUPPL. 1): S78. |
| Wrong intervention | Evaluation of geriatrician-led assessment as prognostic/decision-making aid as to whether to undergo TAVI, but no patient-based geriatric interventions. | Pressler A, Lechner B, Christle J, Bleiziffer S, Mehilli J, Jochheim D, Hettich I, Halle M. Safety, applicability and outcome of regular exercise training after transcatheter aortic valve implantation: a randomized pilot trial. European journal of preventive cardiology 2015; 22(1 SUPPL. 1): S78. |
| Wrong intervention | No intervention assessed | Hansen TB, Berg SK, Sibilitz KL, et al. Availability of, referral to and participation in exercise-based cardiac rehabilitation after heart valve surgery: Results from the national CopenHeart survey. *Eur J Prev Cardiolog.* 2015;22(6):710-8. doi:10.1177/2047487314536364, 10.1177/2047487314536364 |
| Wrong intervention | Evaluation of patient-based approach as prognostic/decision-making aid as to whether to undergo TAVI, but no patient-based geriatric interventions resulting. | Quadrelli P., Marotta M., Baratta S., et al Can an interdisciplinary patient centred approachfor a pre hospitalization preinterventionalscreening of the TAVI patient improve appropriatepatient selection?: A pilot study. *Eur. J. Cardiovasc. Nurs.* 2017;16(SUPPL 1):S36-S37. doi:10.1177/1474515117700580 |
| Wrong intervention | Dietary assessment only, no patient-based intervention | Van Erck D., Tieland M., Weijs P.J., Scholte-op-Reimer W.J., Henriques J.P., Schoufour J.D. Comprehensive dietary assessment in patients undergoing transcatheter aortic valve implantation. *Clin. Nutr. ESPEN* 2020;40:679. doi:10.1016/j.clnesp.2020.09.827 |
| Wrong intervention | CGA evaluated as an assessment tool only, no patient-based intervention | Menendez-Colino R., Ezponda L.E., Guzman G., et al Comprehensive geriatric and frailty assessment in a sample of older patients with severe aortic stenosis considered as candidates for transcatheter aortic valve implantation. *Eur. Geriatr. Med.* 2019;10(Supplement 1):S84. doi:10.1007/s41999-019-00221-0 |
| Wrong intervention | CGA evaluated as an assessment tool only, no patient-based intervention | Onoro-Algar C., Baeza-Monedero M.E., De Villaumbrosia C.G., et al Comprehensive geriatric assessment in patients with aortic severe stenosis. *Eur. Geriatr. Med.* 2019;10(Supplement 1):S79-S80. doi:10.1007/s41999-019-00221-0 |
| Wrong intervention | Descriptive study of outcomes in patients following TAVI, no patient-based intervention evaluated | Beishuizen S.J., Festen S., Loonstra Y.E., van der Werf H.W., de Rooij S.E., van Munster B.C. Delirium, functional decline and quality of life after transcatheter aortic valve implantation: An explorative study. *Geriatr. Gerontol. Int.* 2020;20(12):1202-1207. doi:10.1111/ggi.14064 |
| Wrong intervention | Evaluating a frailty assessment as a screening tool, no patient-based intervention | Maltagliati A., Shi S.M., Kim D. Development of a practical two-stage frailty assessment for older adults undergoing aortic valve replacement. *J. Am. Geriatr. Soc.* 2019;67(Supplement 1):S3. doi:10.1111/jgs.15898 |
| Wrong intervention | Descriptive study of outcomes in patients following TAVI, no patient-based intervention evaluated - pre-operative CGA performed as assessment only. | Kim D.H., Afilalo J., Shi S.M., et al Evaluation of Changes in Functional Status in the Year after Aortic Valve Replacement. *JAMA Intern. Med.* 2019;179(3):383-391. doi:10.1001/jamainternmed.2018.6738 |
| Wrong intervention | Descriptive study of outcomes in patients following TAVI, no patient-based intervention evaluated | Eder V., Ebner Ch., Koller H., et al Evaluation of changes in geriatric symptoms and MRI- or CT-determined cerebral embolic lesions among patients undergoing transcatheter aortic valve implantation before and after procedure. *J. Kardiol.* 2012;19(5-6):190. Cited in: Embase at http://ovidsp.ovid.com/ovidweb.cgi?T=JS&PAGE=reference&D=emed13&NEWS=N&AN=70801333. Accessed May 03, 2022. |
| Wrong intervention | Descriptive study of outcomes in patients following TAVI, no patient-based intervention evaluated | Roqueta C., Herrero M., Martinez-Fernandez M.I., et al Functional and cognitive status of patients after a transcatheter aortic valve implantation. *Eur. Geriatr. Med.* 2018;9(Supplement 1):S154-S155. doi:10.1007/s41999-018-0097-4 |
| Wrong intervention | Descriptive study of outcomes in patients following TAVI, no patient-based intervention evaluated | Andre Monteiro A., Portugal G., Abreu A., et al Functional capacity, depression, anxiety and frailty assessment of patients undergoing transcatheter versus surgical aortic prosthetic valve implantation. *Eur. J. Heart Fail.* 2014;16(SUPPL. 2):255. doi:10.1002/ejhf.93-18 |
| Wrong intervention | Descriptive study of outcomes in patients following TAVI, no patient-based intervention evaluated | Fukui S., Kawakami M., Hayashida K., et al Functional status and instrumental activities of daily living after transcatheter aortic valve replacement. *Top. Geriatr. Rehabil.* 2021;37(2):128-131. doi:10.1097/TGR.0000000000000313 |
| Wrong intervention | CGA evaluated as an assessment tool only, no patient-based intervention | Rouffiac S., Borz B., Chassagne P., Eltchaninoff H. Geriatric assessment in patients undergoing transcatheter aortic valve implantation. *Arch. Cardiovasc. Dis.* 2012;4(SUPPL. 1):77. Cited in: Embase at http://ovidsp.ovid.com/ovidweb.cgi?T=JS&PAGE=reference&D=emed13&NEWS=N&AN=72017384. Accessed May 03, 2022. |
| Wrong intervention | Evaluation of geriatric assessment as a predictive tool for delirium | van der Wulp K., van Wely M.H., Schoon Y., et al Geriatric assessment in the prediction of delirium and long-term survival after transcatheter aortic valve implantation. *J. Thorac. Cardiovasc. Surg.* 2021;161(6):2095-2102.e3. doi:10.1016/j.jtcvs.2020.02.076 |
| Wrong intervention | No intervention assessed, paper examining predictive value of certain patient characteristics in deciding rehabilitation modality | Eichler S., Voller H., Reibis R., et al Geriatric or cardiac rehabilitation? Predictors of treatment pathways in advanced age patients after transcatheter aortic valve implantation. *BMC Cardiovasc. Disord.* 2020;20(1):no pagination. doi:10.1186/s12872-020-01452-x |
| Wrong intervention | CGA evaluated as an prognostic assessment tool only, no patient-based intervention | Fumagalli C., Nardi G., Demola P., et al Impact of frailty on medium-term follow-up in patients undergoing transcatheter aortic valve implantation in a high flow referral centre with high volumes. *Eur. Heart J. Suppl.* 2021;23(SUPPL G):G70-G71. doi:10.1093/eurheartj/suab134.018 |
| Wrong intervention | Evaluation of EFT as a prognostic tool, no patient-based intervention | Lauck S., Ma T., Achtem L., Arora R., Webb J.G., Afilalo J. Implementation of the essential frailty toolset: Improving treatment decision for transcatheter aortic valve implantation. *Can. J. Cardiol.* 2017;33(10 Supplement 1):S225-S226. Cited in: Embase at http://ovidsp.ovid.com/ovidweb.cgi?T=JS&PAGE=reference&D=emed18&NEWS=N&AN=622993092. Accessed May 03, 2022. |
| Wrong intervention | Descriptive study of outcomes in patients following TAVI, no patient-based intervention evaluated | Tobe A., Tanaka A., Tokuda Y., et al Improvement in the nutritional status after transcatheter aortic valve implantation. *J. Cardiol.* 2021;78(3):250-254. doi:10.1016/j.jjcc.2021.04.006 |
| Wrong intervention | Evaluation of interdisciplinary assessment as prognostic/decision-making aid as to whether to undergo TAVI, but no patient-based geriatric interventions. | Roqueta C., Farre N., Vaquerizo B., et al Interdisciplinary assessment in elderly patients with severe aortic stenosis: Decision making process for transcatheter aortic valve implantation. *Eur. Geriatr. Med.* 2017;8(Supplement 1):S181. Cited in: Embase at http://ovidsp.ovid.com/ovidweb.cgi?T=JS&PAGE=reference&D=emed18&NEWS=N&AN=618531690. Accessed May 03, 2022. |
| Wrong intervention | Evaluation of introduction of an interdisciplinary heart team, rather than comprehensive geriatric assessment | Martinez G.J., Seco M., Jaijee S.K., et al Introduction of an interdisciplinary heart team-based transcatheter aortic valve implantation programme: Short and mid-term outcomes. *Intern. Med. J.* 2014;44(9):876-883. doi:10.1111/imj.12514 |
| Wrong intervention | Multidimensional prognostic index evaluated as prognostic assessment tool only, no patient-based intervention. | Gerloni R., Lardieri G., Carriere C., et al Multidimensional evaluation of elderly patients admitted to a cardiology department: Role of multidimensional prognostic index. *Ital. J. Med.* 2013;7(SUPPL. 2):55. Cited in: Embase at http://ovidsp.ovid.com/ovidweb.cgi?T=JS&PAGE=reference&D=emed14&NEWS=N&AN=71295817. Accessed May 03, 2022. |
| Wrong intervention | Evaluation of geriatrician-led assessment as prognostic/decision-making aid as to whether to undergo TAVI, but no patient-based geriatric interventions. | Acta Cardiologica. Conference: 33^rd^ Annual Scientific Meeting of Belgian Society of Cardiology. (33^rd^). Bruxelles Belgium. Sponsor: AstraZeneca, Bayer Bayer, BIOTRONINK, Boehringer Ingelheim, Boston Scientific, Birstol-Myers Squibb, Medtronic, MSD Be Well, PHILIPS, SANOFI, SERVIER, Pfizer . Conference Publication: (pp. 68-99). 68(1) (pp 81), 2014. Date of Publication: 2014. |
| Wrong intervention | Evaluation of geriatrician-led assessment as prognostic/decision-making aid as to whether to undergo TAVI, but no patient-based geriatric interventions. | Collas V., Chong Y.M., Paelinck B., et al Multidimensional geriatric assessment in the decision for treatment of older severe aortic valve stenosis patients. *Eur. Geriatr. Med.* 2015;6(SUPPL. 1):S67. Cited in: Embase at http://ovidsp.ovid.com/ovidweb.cgi?T=JS&PAGE=reference&D=emed16&NEWS=N&AN=72037289. Accessed May 03, 2022. |
| Wrong intervention | No assessment, only same universal intervention, doesn't fall into scope of CGA | Lauck S., Sathananthan J., Achtem L., Humphries K., Webb J., Wood D. NURSE-LED POST-PROCEDURE PROTOCOL TO FACILITATE SAFE NEXT-DAY DISCHARGE HOME: FINDINGS OF THE 3M TAVR STUDY. *Can. J. Cardiol.* 2019;35(10 Supplement):S204. doi:10.1016/j.cjca.2019.07.385 |
| Wrong intervention | No intervention | Kok C.S.E., Jansen W.M., Van Beek M.W.H. Patient selection for geriatric screening prior to TAVR: A clinical challenge. *Eur. Geriatr. Med.* 2021;12(SUPPL 1):S83. doi:10.1007/s41999-021-00585-2 |
| Wrong intervention | CGA evaluated as prognostic assessment tool only, no patient-based intervention. | Bo M., Bergamo D., Calvi E., et al Role of comprehensive geriatric assessment in low surgical risk older patients with aortic stenosis. *Aging Clin. Exp. Res.* 2020;32(3):381-388. doi:10.1007/s40520-019-01228-0 |
| Wrong Intervention | CGA evaluated as prognostic assessment tool only, no patient-based intervention. | Mannarino G, Van Der Velde N, Thibodeau M-P, Baan J, Van Mourik M, Masson JB, Vis MM, Bramlage P, Kurucova J, Thoenes M, Michel J-P, Schoenenberger A, Ungar A. Transcatheter aortic valve implantation registry with comprehensive geriatric assessment. European geriatric medicine. Conference: 12th international congress of the european union geriatric medicine society, EUGMS 2016. Portugal. Conference start: 20161005. Conference end: 20161007 2016; 7: S15‐S16. |
| Wrong outcomes | No measure of effect of intervention | Roqueta C., Vaquerizo B., Martinez-Fernandez M.I., et al Interventions in frail elderly patients with severe aortic stenosis, possible candidates for implementation of aortic valve transcatheter (TAVI). *Eur. Geriatr. Med.* 2017;8(Supplement 1):S230. Cited in: Embase at http://ovidsp.ovid.com/ovidweb.cgi?T=JS&PAGE=reference&D=emed18&NEWS=N&AN=618532166. Accessed May 03, 2022. |
| Wrong outcomes | CGA evaluated as an prognostic assessment tool (e.g. for length of stay) only, describes interventions implemented by geriatricians but doesn't measure the effect of the CGA interventions implemented. | Bobet A.S., Brouessard C., Le Tourneau T., Manigold T., De Decker L., Boureau A.-S. Length of Stay in Older Patients Undergoing Transcatheter Aortic Valve Replacement: Value of a Geriatric Approach. *Gerontology* 2021;no pagination. doi:10.1159/000518821 |
| Wrong outcomes | Doesn't report valid outcomes measuring effect of intervention | Wong S., Montoya L., Quinlan B. Transitional care post TAVI: A pilot initiative focused on bridging gaps and improving outcomes. *Geriatr Nurs* 2018;39(5):548-553. doi:10.1016/j.gerinurse.2018.03.003 |
| Wrong outcomes | Doesn't report valid outcomes measuring effect of intervention | Damier E, Chidlovskii E, Bertrand B, Dang VM, Vanzetto G, Couturier P. Évaluation gériatrique avant décision de remplacement valvulaire aortique par voie percutanée chez des patients âgés fragiles et suivi à un an : intérêt d'une collaboration cardio-gériatrique ? [Multidimensional geriatric assessment before transcatheter aortic valve implantation in frail elderly patients with one-year follow-up. Cardio-geriatrician collaboration benefits?]. Ann Cardiol Angeiol (Paris). 2016 Sep;65(4):250-4. French. doi: 10.1016/j.ancard.2016.05.001. Epub 2016 Jul 15. PMID: 27427467. |
| Wrong population | Surgical Aortic Valve Replacements only | Porterie J., Kalavrouziotis D., Dumont E., et al Clinical impact of the heart team on the outcomes of surgical aortic valve replacement among octogenarians. *J. Thorac. Cardiovasc. Surg.* 2021;no pagination. doi:10.1016/j.jtcvs.2021.03.030 |
| Wrong population | Patients planned for valve surveillance rather than planned for TAVI | 1. Eddington R, Johnson K. Echo Waitlists can be Significantly Reduced by Determining Patients' Goals of Care (GOC) and Assessing Frailty Utilising a Nurse Led Clinic. Heart, Lung and Circulation. 2021;30:S59-S60. doi:10.1016/j.hlc.2021.05.007 |
| Wrong population | Too broad - includes patients admitted with acute cardiovascular conditions, not only those awaiting TAVI, with no separate TAVI subgroup analysis. Also may include emergency TAVI cases. | Van Grootven B., Jeuris A., Jonckers M., et al Geriatric co-management for cardiology patients in the hospital: A quasi-experimental study. *J. Am. Geriatr. Soc.* 2021;69(5):1377-1387. doi:10.1111/jgs.17093 |
| Wrong population | Too broad - includes all types of valve replacement patients, no subgroup analysis of TAVI patients | Tamuleviciute-Prasciene E., Beigiene A., Thompson M.J., Balne K., Kubilius R., Bjarnason-Wehrens B. The impact of additional resistance and balance training in exercise-based cardiac rehabilitation in older patients after valve surgery or intervention: randomized control trial. *BMC Geriatr* 2021;21(1):23. doi:10.1186/s12877-020-01964-3 |
| Wrong population | Mean +- 1.96 x standard deviation was less than 65 years old | Lin Xu, Jiafu Wei, Jiani Liu, Yuan Feng, Lu Wang, Shiqi Wang, Qiao Li, Sen He, Yong Chen, Yong Peng, Yun Bao, Xuemei Yang, Chengqi He, Mao Chen, Quan Wei, Inspiratory muscle training improves cardiopulmonary function in patients after transcatheter aortic valve replacement: a randomized clinical trial, European Journal of Preventive Cardiology, Volume 30, Issue 2, February 2023, Pages 191–202, https://doi.org/10.1093/eurjpc/zwac269 |
| Wrong study type | Opinion/Review Article | Ungar A., Bramlage P., Thoenes M., Zannoni S., Michel J.-P. A call to action - Geriatricians' experience in treatment of aortic stenosis and involvement in transcatheter aortic valve implantation. *Eur. Geriatr. Med.* 2013;4(3):176-182. doi:10.1016/j.eurger.2013.04.007 |
| Wrong study type | Opinion/Review Article | Eichler S., Voller H. Advances in cardiac rehabilitation: Cardiac rehabilitation after transcatheter aortic valve implantation. *Monaldi Arch. Chest Dis. Card. Ser.* 2016;86(1-2):no pagination. doi:10.4081/monaldi.2016.758 |
| Wrong study type | Protocol | Sola M., Ramm C.J., Kolarczyk L.M., et al Application of a Multidisciplinary Enhanced Recovery After Surgery Pathway to Improve Patient Outcomes After Transcatheter Aortic Valve Implantation. *Am. J. Cardiol.* 2016;118(3):418-423. doi:10.1016/j.amjcard.2016.05.015 |
| Wrong study type | Protocol | NCT02855099 . Can Rehabilitation After TAVI Precipitate Recovery and Improve Prognosis. https://clinicaltrials.gov/show/NCT02855099 2016; |
| Wrong study type | Protocol | Deschodt M., Van Grootven B., Jeuris A., et al Geriatric CO-mAnagement for Cardiology patients in the Hospital (G-COACH): Study protocol of a prospective before-after effectiveness-implementation study. *BMJ Open* 2018;8(10):no pagination. doi:10.1136/bmjopen-2018-023593 |
| Wrong study type | Opinion/Review Article | Andersen T.W. Physical activity during hospitalisation post-TAVI-an important part of patient rehabilitation. *EuroIntervention* 2016;354. Cited in: Embase at http://ovidsp.ovid.com/ovidweb.cgi?T=JS&PAGE=reference&D=emed17&NEWS=N&AN=611934551. Accessed May 03, 2022. |
| Wrong study type | Protocol | NCT03801460 . Physiological Reconditioning Program Administered Remotely in Patients Undergoing Transcatheter Aortic Valve Replacement: A Pilot Study. https://clinicaltrials.gov/show/NCT03801460 2019 |
| Wrong study type | Protocol | NCT02597985 . Prehabilitation to Improve Functional and Clinical Outcomes in Patients With Aortic Stenosis. https://clinicaltrials.gov/show/NCT02597985 201 |
| Wrong study type | Protocol | ChiCTR1900026403 . The Effect of Traditional Chinese Medicine Combined With Cardiac Rehabilitation on Cardiac Function and Quality of Life in Patients after TAVR: a Randomized Controlled Trial. https://trialsearch.who.int/Trial2.aspx?TrialID=ChiCTR1900026403 2019; |
| Wrong study type | Opinion/Review Article | Lantelme P., Harbaoui B. The optimal work-up before TAVI. *Ann. Cardiol. Angeiol.* 2019;68(6):410-414. doi:10.1016/j.ancard.2019.09.016 |
| Wrong study type | Protocol | NCT03522454 . The PERFORM-TAVR Trial. https://clinicaltrials.gov/show/NCT03522454 2018 |
| Wrong study type | Opinion/Review Article | Ungar A., Schoenenberger A., Maggi S., Martinez-Selles M., Michel J.-P. The value of comprehensive geriatric assessment in elderly patients with severe aortic stenosis - A position statement of the European Union Geriatric Medicine Society (EUGMS). *Eur. Geriatr. Med.* 2015;6(3):271-273. doi:10.1016/j.eurger.2014.12.011 |
| Wrong study type | Protocol | DRKS000305267.  Prehabilitation "Karl-Heinz" with a focus on cardiac and cognitive functions prior to interventions on the heart: an analysis of the state of health – PRECOVERY  <https://trialsearch.who.int/Trial2.aspx?TrialID=DRKS00030526> |
| Wrong study type | Protocol | ISRCTN32148715  Comparative evaluation of cardiovascular rehabilitation programs in patients after transcutaneous aortic valve replacement; with evaluation of ergospirometric, functional, imaging and biochemical data and markers  https://trialsearch.who.int/Trial2.aspx?TrialID=ISRCTN32148715 |
| Wrong study type | Protocol | NCT05989594  Home-based Mobile Guided Exercise-based Cardiac Rehabilitation Among Patients Undergoing TAVR (REHAB-TAVR)  <https://clinicaltrials.gov/ct2/show/NCT05989594> |
| Wrong study type | Conference abstract | James Harvey, Paul H. Tolerico, Theodore Bell, Lara Mason, Heather McKinney et al.; Prehabilitation for patients undergoing Transcatheter Aortic Valve Replacement: a pilot randomized clinical trial. Journal of the American College of Cardiology. 2023.  <https://doi.org/10.1016/S0735-1097(23)01312-8>. |
| Wrong study type | Conference abstract | Daniela Wong Pacheco, Victor Silva Escobedo, Su-Jau Yang, Michael Najem, Jennifer L. Nguyen, Kristi Reynolds, Nancy Gin et al. Comparison of home-based vs center-based cardiac rehabilitation in post-TAVR patients. Journal of the American College of Cardiology. 2023  https://doi.org/10.1016/S0735-1097(23)02799-7. |
| Wrong study type | Protocol | Shen Z, Mi S, Huang C, Zhou D, Pan W, Xu X, Lin Y, Zhang Y. Home-based mobile-guided exercise-based cardiac rehabilitation among patients undergoing transcatheter aortic valve replacement (REHAB-TAVR): protocol for a randomised clinical trial. BMJ Open. 2024 Mar 7;14(3):e080042. doi: 10.1136/bmjopen-2023-080042. PMID: 38453208; PMCID: PMC10921510. |
| Wrong study type | Review article | Zou J, Yuan J, Liu J, Geng Q. Impact of cardiac rehabilitation on pre- and post-operative transcatheter aortic valve replacement prognoses. Front Cardiovasc Med. 2023 Dec 13;10:1164104. doi: 10.3389/fcvm.2023.1164104. PMID: 38152609; PMCID: PMC10751363. |
| Wrong study type | Conference abstract and protocol | Rosie Fountotos et al.Protein and Exercise to Reverse Frailty in Older Men and Women Undergoing Transcatheter Aortic Valve Replacement: Design of the PERFORM-TAVR Trial, Canadian Journal of Cardiology, Volume 40, Issue 2,2024,Pages 267-274,  https://doi.org/10.1016/j.cjca.2023.11.037. |
| Wrong study type | Conference abstract | Abstract 17900: Target Trial Emulation: Evaluating Cardiac Rehabilitation After TAVR  Merilyn Varghese, Issa Dahabreh, Yang Song, Jiaman Xu, Alexis L Beatty, Kevin McConeghy, Robert W Yeh, Laurence Sperling, Gregg C Fonarow, Steven J Keteyian, Wen-Chih H Wu, Dhruv S Kazi <https://doi.org/10.1161/circ.148.suppl_1.17900> |
| Wrong study type | Conference abstract | S Lauck, C Bancroft, M Yu, J Polderman, H Andrews, A Stephenson, Implementation of nurse-led early mobilisation after transcatheter aortic valve implantation: Identification of barriers to standardised practice, European Journal of Cardiovascular Nursing, Volume 21, Issue Supplement_1, July 2022, zvac060.027, https://doi.org/10.1093/eurjcn/zvac060.027 |
| Wrong study type | Protocol | RESTORE-TAVI Pilot  https://clinicaltrials.gov/study/NCT06121921 |
