# Supplementary material for: Comprehensive geriatric assessment, and related interventions, to improve outcomes for older patients undergoing transcatheter aortic valve implantation (TAVI): a systematic review
Source: Eur Geriatr Med. 2024 Sep 27;15(6):1615–30. doi: 10.1007/s41999-024-01035-5 (PMC11631815; doi:10.1007/s41999-024-01035-5)
Supplement: Supplementary file 4 — Supplementary file4 (DOCX 47 kb) [file 41999_2024_1035_MOESM4_ESM.docx]

Appendix 4 - Risk of bias decision rationale

| **Rogers et al, 2018** | | |
| --- | --- | --- |
| **Type of Bias** | **Author’s Judgement (Low Risk/High Risk/Some Concerns)** | **Support for Judgement** |
| Bias arising from the **randomization process** (selection process) | Some concerns | 1.1 Yes.  1.2 Yes.  1.3 Probably yes.  Good randomisation process: “Patients were randomly allocated … in a 1:1 ratio with allocation administered by an investigator independent of the study, stratified by age and gender.”  However, the authors also note: “there was some evidence of imbalance in baseline outcome scores… most likely due to the play of chance in small groups.” But no P values for this are given.  Additionally, 1 patient chose to switch to Standard of Care, and one patient was offered and accepted CR at a difference centre. |
| Bias due to **deviations from intended interventions** (performance bias) | Some concerns | 2.1 Yes.  2.2 Yes.  2.3 Yes.  2.4 Probably yes.  2.5 Yes.  2.6 Probably yes.  2.7 Probably no.  Deviations from intended intervention occurred:  “Following randomisation, one participant in each group switched to the other arm through patient choice.”  There may have been further deviations due to the non-blinded nature of the study. The authors note that the “CR team were often asked about medical management such as clarifying drug regimens and where necessary, opportunistic scheduling of clinic appointments were facilitated potentially reducing admission rates.”  However, the authors used an appropriate analysis method: “Patient reported outcomes for both groups were reported descriptively according to intention-to-treat principle.” |
| Bias due to **missing outcome data** (attrition bias) | Some concerns | 3.1 No.  3.2 No.  3.3 Probably yes.  3.4 Probably no.  Data not available for all outcomes: “Only 14 of the study patients underwent the 6MWT due to frailty.”  However, the authors state that all participants were monitored and none lost to follow up: “We were able to retain all control and intervention participants over the 6-months of the study and no patients were lost to follow”. |
| Bias in **measurement of the outcome** (detection bias) | Low | 4.1 No.  4.2 Probably no.  4.3 Probably no.  4.4 NA  4.5 NA  “Outcomes were measured…by an assessor blinded to group allocation.” |
| Bias in **selection of the reported result** (reporting bias) | Some concerns | 5.1 No.  5.2 No.  5.3 No.  Trial registered (albeit retrospectively, on Oct 3^rd^ 2016, patients recruited Jun 2016 – Feb 2017) 7on NCT.gov  <https://clinicaltrials.gov/ct2/show/NCT02921880>  Outcomes described differ slightly from those in the protocol. |
| Overall risk of bias judgement | Some concerns |  |

| **Pressler et al, 2016** | | |
| --- | --- | --- |
| **Type of Bias** | **Author’s Judgement (Low Risk/High Risk/Some Concerns)** | **Support for Judgement** |
| Bias arising from the **randomization process** (selection process) | Low | 1.1 Yes.  1.2 Yes.  1.3 No.  Good randomisation process: “Patients were randomly allocated 1:1 according to a computer-generated allocation program code.” |
| Bias due to **deviations from intended interventions** (performance bias) | Some concerns | 2.1 Yes.  2.2 Yes.  2.3 No.  2.4 NA  2.5 NA  2.6 No.  2.7 Probably no.  Non-blinded study. Some variability in the interventions received however likely falls within scope of ‘normal practice’:  “Each patient was motivated to adhere to the basic protocol, but individual adjustments were allowed in case of slower progress.”  “All participants in TG were able to fully adhere to the increasing intensities and durations provided in the protocol”  The authors used a per-protocol analysis however they did state the following: “There is inherent bias in a per-protocol analysis, but applying an intention-to-treat analysis to the primary end point (imputing baseline values of the dropouts for missing values) did not change the results.” |
| Bias due to **missing outcome data** (attrition bias) | Low | 3.1 Probably no.  3.2 Yes.  “Low number of dropouts that were all unrelated to the intervention” – accident, ICH, not willing to continue participation. |
| Bias in **measurement of the outcome** (detection bias) | Some concerns | 4.1 No.  4.2 No.  4.3 Probably yes.  4.4 Probably yes.  4.5 Probably no.  Outcome assessors were blinded: “Experienced medical staff blinded to group assignment.”  However, subjective outcomes measured through questionnaires were subject to bias as non-blinded participants. |
| Bias in **selection of the reported result** (reporting bias) | Low | 5.1 Yes.  5.2 No.  5.3 No.  Endpoints and outcome measures well described in methods section.  “No changes to methods or eligibility criteria were made after study commencement.”  Unusual to report specific KCCQ domains however overall effect for KCCQ as a whole also reported. |
| Overall risk of bias judgement | Some concerns |  |

| **Lindman et al, 2021** | | |
| --- | --- | --- |
| **Type of Bias** | **Author’s Judgement (Low Risk/High Risk/Some Concerns)** | **Support for Judgement** |
| Bias arising from the **randomization process** (selection process) | Some concerns | 1.1 Probably yes.  1.2 No information.  1.3 No.  “[Participants] were randomised 1:1 to an intervention or control group”  “Randomisation was stratified by sex and 5m walk time at the 30 day post-TAVR visit”  However, method of randomisation not specified so unable to comment on allocation sequence concealment. |
| Bias due to **deviations from intended interventions** (performance bias) | Some concerns | 2.1 Probably yes.  2.2 Yes.  2.3 Probably yes.  2.4 Probably yes.  2.5 Yes.  2.6 Yes.  2.7 NA  Control group having an awareness of activity being monitored may have affected their activity habits (i.e. inflated, rather than acting as a pure control group).  Appropriate analysis used: intention-to-treat analysis and pre-specified sub-group analysis. |
| Bias due to **missing outcome data** (attrition bias) | Low | 3.1 Yes.  3.2 NA  3.3 NA  3.4 NA  Missing data reported in Table 2. Data available for nearly all participants randomised. No patients lost to follow up. |
| Bias in **measurement of the outcome** (detection bias) | Some concerns | 4.1 No.  4.2 No.  4.3 Yes.  4.4 Probably yes.  4.5 Probably no.  Standardised commercial watches used.  Researchers non-blinded, participants semi-blinded. |
| Bias in **selection of the reported result** (reporting bias) | Low | 5.1 Probably yes.  5.2 No.  5.3 No.  Primary and secondary endpoints described fully in method; however, not all secondary endpoints discussed in results.  Sub-group analysis performed (of patients who were compliant with study activities but did not enrol in any sort of CR program), but this was pre-specified. |
| Overall risk of bias judgement | Some concerns |  |

| **Weber et al 2021** | | |
| --- | --- | --- |
| **Type of Bias** | **Author’s Judgement (Low Risk/High Risk/Some Concerns)** | **Support for Judgement** |
| Bias arising from the **randomization process** (selection process) | Low | 1.1 Yes.  1.2 Yes.  1.3 No.  Good randomisation process: “Patients were randomised by using a computer-based program in a 1:1 ratio between one of the two treatment groups.”  Appropriate analysis of baseline characteristics performed:  “Baseline characteristics are shown in Table 1 and showed no significant differences between the groups.” |
| Bias due to **deviations from intended interventions** (performance bias) | Some concerns | 2.1 Yes.  2.2 Yes.  2.3 No.  2.4 NA  2.5 NA  2.6 No information.  2.7 Probably no.  Non-blinded study, could lead to some bias in the delivery of the physiotherapy to each group. For example, physiotherapists being more motivated to push patients who are in the intervention group. Participants feeling more encouraged to take part. |
| Bias due to **missing outcome data** (attrition bias) | Low | 3.1 Yes.  3.2 NA.  3.3 NA.  3.4 NA.  Outcomes reported for all patients. |
| Bias in **measurement of the outcome** (detection bias) | Low | 4.1 No.  4.2 No.  4.3 Yes.  4.4 Probably no.  4.5 NA.  Not specified whether outcome assessors blinded in to intervention group, however given they are relatively “hard” outcomes e.g. mortality, unlikely to have significantly influenced measurement of the outcome. |
| Bias in **selection of the reported result** (reporting bias) | Some concerns | 5.1 No information.  5.2 No.  5.3 No.  Not specified in full text report whether outcomes were pre-specified, no link available to protocol. Trial was prospective and approved by local ethics committee so outcome measurements were likely pre-determined but not available. |
| Overall risk of bias judgement | Some concerns |  |

| **Edwards et al, 2020** | | |
| --- | --- | --- |
| **Type of Bias** | **Author’s Judgement (Low Risk/High Risk/Some Concerns)** | **Support for Judgement** |
| Bias arising from the **randomization process** (selection process) | Low | 1.1 Yes.  1.2 Yes.  1.3 No.  Good randomisation process: "We randomized participants 1:1 to receive TAU or the CBT intervention  using a random sequence from <http://www.randomnumbergenerator.org>”  Authors appropriately analysed baseline characteristics: “There were no statistically significant differences between groups on baseline medical or psychiatric variables” |
| Bias due to **deviations from intended interventions** (performance bias) | Some concerns | 2.1 Yes.  2.2 Yes.  2.3 Yes.  2.4 Probably no.  2.5 NA  2.6 Yes.  Non-blinded study vulnerable to bias.  Some heterogeneity in intervention received as some were performed as telephone follow-up rather than face to face.  Appropriate analysis used:  “Our primary analysis followed a modified intention-to-treat principle.”  “All reported outcome data at all time points were used in the analysis, no cases were excluded due to partially missing data at differing time points.” |
| Bias due to **missing outcome data** (attrition bias) | High | 3.1 No.  3.2 No.  3.3 Yes.  3.4 Yes.  Significant amount of missing data: 19 patients lost to follow up in each group due to shortage of research staff.  Authors acknowledge that missingness of data was likely dependent on its true value: “Missing 1 month follow up data among patients who had been depressed at baseline prevented us from analysing the impact of the CBT intervention in this important subsample.” |
| Bias in **measurement of the outcome** (detection bias) | High | 4.1 Yes.  4.2 No.  4.3 NA.  4.4 NA.  4.5 NA.  Inappropriate method of measuring the outcome:  “…the current study was also not designed to detect effect modification and was limited in detecting clinical change. Given these limitations, data from the current study are best considered as hypothesis generating.”  Study not designed to detect effect modification i.e. natural remission of symptoms, only detected clinical change. Possible ceiling effect whereby many of the participants started with subclinical scores on the BDI-II and STAI-Y1.  Self-reported measures used, with non-blinded study participants. This may have influenced their self-reported assessments. |
| Bias in **selection of the reported result** (reporting bias) | Low | 5.1 Yes.  5.2 No.  5.3 No.  Trial Protocol (NCT03798548)  All outcomes that were pre-specified in the trial protocol have been reported. |
| Overall risk of bias judgement | High |  |

| **Vitez et al, 2023** | | |
| --- | --- | --- |
| **Type of Bias** | **Author’s Judgement (Low Risk/High Risk/Some Concerns)** | **Support for Judgement** |
| Bias arising from the **randomization process** (selection process) | Some concerns | 1.1 Yes.  1.2 Probably yes.  1.3 Probably yes.  There was an adequate randomization process.  “Randomization was performed by the recruiting investigator (who was not the recruiting/treating physician to ensure concealed allocation) using adaptive (urn) randomization with sealed envelopes” |
| Bias due to **deviations from intended interventions** (performance bias) | High | 2.1 Yes.  2.2 Yes.  2.3 No.  2.4 Not applicable.  2.5 Not applicable.  2.6 No information.  2.7 Probably yes.  This was a non-blinded study.  The authors did not state whether they used intention-to-treat, per-protocol, or as-treated analyses of the results. There is the potential that this may have impacted the result. |
| Bias due to **missing outcome data** (attrition bias) | High | 3.1 No.  3.2 Probably no.  3.3 Yes.  3.4 Probably yes.  41% of participants did not complete left the study between randomisation and the end of the study resulting in a large amount of lost follow up. They did not state how they addressed the missing data. |
| Bias in **measurement of the outcome** (detection bias) | High | 4.1 No.  4.2 No.  4.3 No information.  4.4 Yes.  4.5 Probably yes.  There is no statement regarding whether the outcome assessors were blinded. The participants were not blinded therefore their answers may have influenced by the knowledge of the intervention received. It is possible that this may have meant the outcome was influenced by this knowledge. |
| Bias in **selection of the reported result** (reporting bias) | High | 5.1 No information.  5.2 No.  5.3 Yes.  There was no statement about the study protocol or what outcomes were pre-specified to be reported.  In the method for statistical analysis the authors stated that they would adjust for BMI. |
| Overall risk of bias judgement | High |  |

| **Hu et al. 2023** | | |
| --- | --- | --- |
| **Type of Bias** | **Author’s Judgement (Low Risk/High Risk/Some Concerns)** | **Support for Judgement** |
| Bias arising from the **randomization process** (selection process) | Low | - 1. Probably yes.   2. Probably yes.   3. No.   Randomisation process was judged to be adequate. The authors stated that “the sealed envelopes were used to assign participants in a 1:1 ratio to the [intervention] group or the guideline control group]  No statistically significant differences in the baseline characteristics were detected. |
| Bias due to **deviations from intended interventions** (performance bias) | Some concerns | 2.1 Yes.  2.2 Yes.  2.3 No information.  2.4 Not applicable.  2.5 Not applicable.  2.6 Yes.  2.7 Not applicable  Participants were not blinded, however it was not possible to blind due to the nature of the intervention.  Data analysis was based on an intention-to-treat principle. |
| Bias due to **missing outcome data** (attrition bias) | Low | 3.1 Probably yes.  It appears that there was little missing data. Intention-to-treat analysis was used. |
| Bias in **measurement of the outcome** (detection bias) | Low | 4.1 No.  4.2 No.  4.3 No.  The assessors were blinded to participant randomisation. |
| Bias in **selection of the reported result** (reporting bias) | Low | 5.1 Probably yes.  5.2 No.  5.3 Probably no.  Statistical analysis plan stated in the methods and the study protocol. This plan appears to have been followed. |
| Overall risk of bias judgement | Some concerns |  |

| **Durand et al. 2024** | | |
| --- | --- | --- |
| **Type of Bias** | **Author’s Judgement (Low Risk/High Risk/Some Concerns)** | **Support for Judgement** |
| Bias arising from the **randomization process** (selection process) | Low | - 1. Yes.   2. Probably yes.   3. No information.   This was a cluster, randomised, controlled study. From the study methods “randomisation was computer-generated and carried out all at once after all participating centres had been identified.”  The authors did not state the p-values for the difference in baseline characteristics between the intervention and control group. |
| Bias due to **deviations from intended interventions** (performance bias) | Some concerns | - 1. Probably yes.   2. Probably yes.   3. No information.   4. Not applicable.   5. Not applicable.   6. No information.   7. No information.   No information given regarding blinding of the participants, carers, or people delivering the intervention. |
| Bias due to **missing outcome data** (attrition bias) | High | 3.1 No information.  3.2 Probably no.  3.3 Probably yes.  3.4 No information.  There was no information about whether there was missing outcome data, why there was missing data, or how they managed missing data. |
| Bias in **measurement of the outcome** (detection bias) | Some concerns | 4.1 No.  4.2 Probably no.  4.3 Probably yes.  4.4 No information.  4.5 Probably no.  There was no information given about whether the outcome assessors were blinded. |
| Bias in **selection of the reported result** (reporting bias) | Some concerns | 5.1 No information  5.2 Probably no.  5.3 Probably no.  There was no published protocol linked to the manuscript. |
| Overall risk of bias judgement | High |  |

**ROBINS-I tool** was used for the non-randomised studies using the following prompting questions:

| **Genta et al, 2017 – Non-randomised, prospective observational study (ROBINS-I)** | | |
| --- | --- | --- |
| **Type of Bias** | **Author’s Judgement (Low / Moderate / Serious / Critical)** | **Support for Judgement** |
| Bias arising from **confounding** | Serious | 1.1 Yes.  1.2 No.  1.3 NA.  1.4 No.  1.5 NA.  1.6 No.  1.7 NA.  1.8 NA.  With respect to assessing the degree of benefit this intervention has for the population of interest, one would expect a degree of improvement in exercise tolerance in a proportion of the patients with severe aortic stenosis in the weeks following valve replacement. This is a major confounding issue.  There was no control group of patients who had valve replacement but no cardiac rehabilitation, therefore flawed to attribute all improvement to cardiac rehabilitation.  With respect to using the sAVR patients as a “control group”, reasonable to use them as a benchmark for comparison of safety and adherence outcomes, however not a valid control group for assessing efficacy of intervention (cardiac rehab) as they have also received this.  The SAVR group and TAVI group had statistically significant differences in baseline characteristics.  “Compared with sAVR, the TAVI group was older and had a higher proportion of coronary heart diseases … and prior myocardial infarction … Comorbidities (evaluated with CIRS-CI) were significantly more present in the TAVI group… CKD … and … COPD … were more prevalent in TAVI; … Logistic EuroSCORE was higher in TAVI than in sAVR patients…” |
| Bias due to **selection of participants into the study** | Moderate | 2.1 No  2.2 NA.  2.3 NA.  2.4 Yes.  2.5 NA.  “Consecutive” TAVI/SAVR patients were selected and there was reasonable use of an age cut off to make populations more comparable.  However; following a discussion between reviewers, we felt the paper lacks pre-specified details on referral criteria for cardiac rehab (referral based on opinion of referring hospital clinical team) and there is, therefore, a risk of bias in the selection of patients to take part in CR following their TAVI or SAVR procedure. |
| Bias due to **classification of interventions** | Low | 3.1 Yes.  3.2 Yes.  3.3 No.  Clearly defined intervention  “All patients underwent a 3-week intensive, supervised, tailored aerobic incremental exercise training program.” |
| Bias due to **deviations from intended interventions** | Low | 4.1 No.  4.2 NA.  Slight non-uniformity in the intervention received: “In patients who were  bedridden or markedly disabled or with a high risk of falls at admission, we started with individualized physical therapy interventions for early mobilization” however this would fall under the realms of usual practice |
| Bias due to **missing outcome data** (attrition bias) | Moderate | 5.1 No.  5.2 No.  5.3 No.  5.4 No.  5.5 No.  The authors acknowledge a discrepancy in the amount of missing data between the intervention and control groups:  “Two SAVR and seven TAVI patients had complications”  “A larger percentage of patients after TAVI were unable to perform the 6MWT” |
| Bias in **measurement of the outcome** (detection bias) | Moderate | 6.1 Probably yes.  6.2 Yes.  6.3 Yes.  6.4 No.  Non-blinded prospective study; however, outcomes were measured using reliable and valid tests, delivered by trained staff. |
| Bias in **selection of the reported result** (reporting bias) | Moderate | 7.1 No.  7.2 No.  7.3 No.  No evidence of selective reporting of outcomes; however, pre-specified plan/protocol not available. |
| **Overall risk of bias judgement** | Serious |  |

| **Russo et al. 2014 Non-randomised observational study. Study design not specified as retrospective vs prospective. (ROBINS-I)** | | |
| --- | --- | --- |
| **Type of Bias** | **Author’s Judgement (Low / Moderate / Serious / Critical)** | **Support for Judgement** |
| Bias arising from **confounding** | Serious | 1.1 Yes.  1.2 No.  1.3 NA.  1.4 No.  1.5 NA.  1.6 No.  1.7 NA.  1.8 NA.  With respect to assessing the degree of benefit this intervention has for the population of interest, one would expect a degree of improvement in exercise tolerance in a proportion of the patients with severe aortic stenosis in the weeks following valve replacement. This is a major confounding issue.  There was no control group of patients who had valve replacement but no cardiac rehabilitation, therefore flawed to attribute all improvement to cardiac rehabilitation.  With respect to using the sAVR patients as a “control group”, reasonable to use them as a benchmark for comparison of safety and adherence outcomes, however not a valid control group for assessing efficacy of intervention (cardiac rehab) as they have also received this.  The SAVR group and TAVI group had statistically significant differences in baseline characteristics.  “As expected, the TAVI group  showed more comorbidities and a higher prevalence of left bundle branch block and new pacemaker implantation” |
| Bias due to **selection of participants into the study** | Moderate | 2.1 No  2.2 NA.  2.3 NA.  2.4 Yes.  2.5 NA.  “Consecutive” TAVI/SAVR patients were selected.  However; following a discussion between reviewers, we felt the paper lacks pre-specified details on referral criteria for cardiac rehab (referral based on opinion of referring hospital clinical team) and there is, therefore, a risk of bias in the selection of patients to take part in CR following their TAVI or SAVR procedure. |
| Bias due to **classification of interventions** | Low | 3.1 Yes.  3.2 Yes.  3.3 No.  Clearly defined intervention groups. |
| Bias due to **deviations from intended interventions** | Low | 4.1 No.  4.2 NA. |
| Bias due to **missing outcome data** (attrition bias) | Moderate | 5.1 Probably no.  5.2 No.  5.3 No.  5.4 No information.  5.5 No.  “A 6-min walking test was performed at entry…in 82% of TAVI patients vs. 92% of sAVR patients”  “In 61% of TAVI and 75% of s AVR patients, a CPET test could also be performed before discharge”  Outcome results are at risk of being skewed by those with poor functional ability being unable to complete tests and data for these participants being missing as a result. |
| Bias in **measurement of the outcome** (detection bias) | Low | 6.1 Probably no.  6.2 Yes.  6.3 Yes.  6.4 No.  Non-blinded study; however, objective outcome measures were used and we felt that the outcome assessors being aware of the intervention received most likely did not confer any significant risk of bias. |
| Bias in **selection of the reported result** (reporting bias) | Moderate | 7.1 No.  7.2 No.  7.3 No.  No evidence of selective reporting of outcomes; however, pre-specified plan/protocol not available. |
| **Overall risk of bias judgement** | Serious |  |

| **Imran et al 2018 – Non-randomised retrospective observational study. (ROBINS-I)** | | |
| --- | --- | --- |
| **Type of Bias** | **Author’s Judgement (Low / Moderate / Serious / Critical)** | **Support for Judgement** |
| Bias arising from **confounding** | Serious | 1.1 Yes.  1.2 No.  1.3 NA.  1.4 No.  1.5 NA.  1.6 No.  1.7 NA.  1.8 NA.  With respect to assessing the degree of benefit this intervention has for the population of interest, one would expect a degree of improvement in exercise tolerance in a proportion of the patients with severe aortic stenosis in the weeks following valve replacement. This is a major confounding issue.  There was no control group of patients who had valve replacement but no cardiac rehabilitation, therefore flawed to attribute all improvement to cardiac rehabilitation.  With respect to using the sAVR patients as a “control group”, reasonable to use them as a benchmark for comparison of safety and adherence outcomes, however not a valid control group for assessing efficacy of intervention (cardiac rehab) as they have also received this.  The SAVR group and TAVI group had statistically significant differences in baseline characteristics.  “Compared to SAVR patients, TAVR patients were older, and more likely to be white and to have diabetes, hypertension, dyslipidaemia, cerebrovascular disease, chronic kidney disease and lower functional capacity and exercise duration at baseline.”  There are likely to be other confounding variables that have not been controlled for. |
| Bias due to **selection of participants into the study** | Moderate | 2.1 Yes.  2.2 Probably yes.  2.3 Probably yes.  2.4 Probably yes.  2.5 No.  Authors have reported the proportion of patients referred, and enrolled, in cardiac rehabilitation in each group.  However, “patients who participated in 10 or less exercise sessions were considered lost to follow-up and excluded from the final analysis.” |
| Bias due to **classification of interventions** | Low | 3.1 Yes  3.2 Yes.  3.3 No.  Low ambiguity in intervention groups and nature of intervention. |
| Bias due to **deviations from intended interventions** | Low | 4.1 No  4.2 NA.  No reported cases of modification or discontinuation of intervention. |
| Bias due to **missing outcome data** (attrition bias) | Moderate | 5.1 No.  5.2 No.  5.3 Yes.  5.4 No.  No information on missing data for each individual outcome measure but proportions of and reasons  for missing participants differ  slightly across intervention  groups (significant number of patients dropped out of SAVR group (11/102)).  Participants were excluded from the final analysis due to missing data: “patients who participated in 10 or less exercise sessions were considered lost to follow-up and excluded from the final analysis.” |
| Bias in **measurement of the outcome** (detection bias) | Low | 6.1 Probably no.  Retrospective  6.2 Yes.  6.3 Probably yes.  6.4 No.  Retrospective analysis, so participants and assessors unlikely to have knowledge of involvement in study at time measurements made. Exercise duration and intensity objective measures (though potentially motivated by intrinsic factors surrounding patient’s motivation). |
| Bias in **selection of the reported result** (reporting bias) | Moderate | 7.1 No.  7.2 No.  7.3 No.  No evidence of selective reporting of outcomes; however, pre-specified plan/protocol not available. |
| **Overall risk of bias judgement** | Serious |  |

| **Voller et al 2015 – Non-randomised retrospective observational study. (ROBINS-I)** | | |
| --- | --- | --- |
| **Type of Bias** | **Author’s Judgement (Low / Moderate / Serious / Critical)** | **Support for Judgement** |
| Bias arising from **confounding** | Serious | 1.1 Yes.  1.2 No.  1.3 NA.  1.4 No.  1.5 NA.  1.6 No.  1.7 NA.  1.8 NA.  With respect to assessing the degree of benefit this intervention has for the population of interest, one would expect a degree of improvement in exercise tolerance in a proportion of the patients with severe aortic stenosis in the weeks following valve replacement. This is a major confounding issue.  There was no control group of patients who had valve replacement but no cardiac rehabilitation, therefore flawed to attribute all improvement to cardiac rehabilitation.  With respect to using the sAVR patients as a “control group”, reasonable to use them as a benchmark for comparison of safety and adherence outcomes, however not a valid control group for assessing efficacy of intervention (cardiac rehab) as they have also received this.  Analysis showed significant baseline differences between the TAVI and SAVR groups. The authors made attempts to control for these variables through statistical analysis; however, there are likely to be other confounding variables that have not been controlled for. |
| Bias due to **selection of participants into the study** | Moderate | 2.1 No  2.2 NA.  2.3 NA.  2.4 Yes.  2.5 NA.  “Consecutive” TAVI/SAVR patients were selected.  However; following a discussion between reviewers, we felt the paper lacks pre-specified details on referral criteria for cardiac rehab (referral based on opinion of referring hospital clinical team) and there is, therefore, a risk of bias in the selection of patients to take part in CR following their TAVI or SAVR procedure. |
| Bias due to **classification of interventions** | Low | 3.1 Yes.  3.2 Yes.  3.3 No.  Low ambiguity in intervention groups and nature of intervention. |
| Bias due to **deviations from intended interventions** | Low | 4.1 No.  4.2 NA.  No reported cases of modification or discontinuation of intervention. |
| Bias due to **missing outcome data** (attrition bias) | Moderate | 5.1 No information.  5.2 No.  5.3 No.  5.4 NA.  5.5 NA.  Not specified whether the outcome data reported for all patients (seems unlikely that all 442 participants were able to complete cardiac rehabilitation and have all functional / emotional outcomes measured without complication). Possibility of only including patients who completed the rehab program and for whom outcomes were available (which would introduce selection bias). |
| Bias in **measurement of the outcome** (detection bias) | Low | 6.1 Probably no.  6.2 Yes.  6.3 Probably yes.  6.4 No.  Standardised method described in methods section.  Retrospective analysis, so participants and assessors unlikely to have knowledge of involvement in study at time measurements made. |
| Bias in **selection of the reported result** (reporting bias) | Moderate | 7.1 No.  7.2 No.  7.3 No.  No pre-specified plan/protocol available and as data collection and analysis was conducted retrospectively, authors could have chosen outcomes in the knowledge of whether any significant effect was demonstrated.  However, there is no evidence of selective reporting of outcomes and there is a broad range of outcomes reported with both positive and negative findings. |
| **Overall risk of bias judgement** | Serious |  |

| **Fauchere et al 2014 - Non-randomised retrospective cohort study. (ROBINS-I)** | | |
| --- | --- | --- |
| **Type of Bias** | **Author’s Judgement (Low / Moderate / Serious / Critical)** | **Support for Judgement** |
| Bias arising from **confounding** | Serious | 1.1 Yes.  1.2 No.  1.3 NA.  1.4 No.  1.5 NA.  1.6 No.  1.7 NA.  1.8 NA.  With respect to assessing the degree of benefit this intervention has for the population of interest, one would expect a degree of improvement in exercise tolerance in a proportion of the patients with severe aortic stenosis in the weeks following valve replacement. This is a major confounding issue.  There was no control group of patients who had valve replacement but no cardiac rehabilitation, therefore flawed to attribute all improvement to cardiac rehabilitation.  With respect to using the sAVR patients as a “control group”, reasonable to use them as a benchmark for comparison of safety and adherence outcomes, however not a valid control group for assessing efficacy of intervention (cardiac rehab) as they have also received this.  Analysis showed significant variance in baseline characteristics between two groups, therefore poor control group and significant risk of confounding bias. |
| Bias due to **selection of participants into the study** | Moderate | 2.1 No  2.2 NA.  2.3 NA.  2.4 Yes.  2.5 NA.  “Consecutive” TAVI/SAVR patients were selected.  However; following a discussion between reviewers, we felt the paper lacks pre-specified details on referral criteria for cardiac rehab (referral based on opinion of referring hospital clinical team) and there is, therefore, a risk of bias in the selection of patients to take part in CR following their TAVI or SAVR procedure.  Dataset appears to be complete for entire group of 112 included participants.  Unlikely that all participants completed all outcome measures but this is not specified.  Given retrospective study design, possible only patients who successfully completed cardiac rehabilitation were included (this would lead to selection bias.) |
| Bias due to **classification of interventions** | Low | 3.1 Yes.  3.2 Yes.  3.3 No.  Low ambiguity in intervention groups and nature of intervention. |
| Bias due to **deviations from intended interventions** | Low | 4.1 No.  4.2 NA.  No reported cases of modification or discontinuation of intervention. |
| Bias due to **missing outcome data** (attrition bias) | Moderate | 5.1 No information.  5.2 No.  5.3 No.  5.4 NA.  5.5 NA.  Dataset appears to be complete for entire group of 112 included participants.  Unlikely that all participants completed all outcome measures but this is not specified.  Given retrospective study design, possible only patients who successfully completed cardiac rehabilitation were included (this would lead to selection bias.) |
| Bias in **measurement of the outcome** (detection bias) | Low | 6.1 Probably no.  6.2 Yes.  6.3 Probably yes.  6.4 No.  Retrospective analysis, so participants and assessors unlikely to have knowledge of involvement in study at time measurements made. |
| Bias in **selection of the reported result** (reporting bias) | Moderate | 7.1 No.  7.2 No.  7.3 No.  No pre-specified plan/protocol available and as data collection and analysis was conducted retrospectively, authors could have chosen outcomes in the knowledge of whether any significant effect was demonstrated.  However, there is no evidence of selective reporting of outcomes and there is a broad range of outcomes reported with both positive and negative findings. |
| **Overall risk of bias judgement** | Serious |  |

| **Zanettini et al 2014 – Non-randomised prospective observational study. (ROBINS-I)** | | |
| --- | --- | --- |
| **Type of Bias** | **Author’s Judgement (Low / Moderate / Serious / Critical)** | **Support for Judgement** |
| Bias arising from **confounding** | Serious | 1.1 Yes.  1.2 No.  1.3 NA.  1.4 No.  1.5 NA.  1.6 No.  1.7 NA.  1.8 NA.  With respect to assessing the degree of benefit this intervention has for the population of interest, one would expect a degree of improvement in exercise tolerance in a proportion of the patients with severe aortic stenosis in the weeks following valve replacement. This is a major confounding issue.  There was no control group of patients who had valve replacement but no cardiac rehabilitation, therefore flawed to attribute all improvement to cardiac rehabilitation. |
| Bias due to **selection of participants into the study** | Moderate | 2.1 No.  2.2 NA.  2.3 NA.  2.4 Probably no.  2.5 No.  Some degree of uncertainty in how patients were selected to take part. Needing 6 months follow up creates potential for selection bias with regard to geographic location/functional ability. No eligibility criteria reported, authors simply state “we limited enrolment into this study to patients sent by this high-volume center because we were asked to participate in the follow-up.” |
| Bias due to **classification of interventions** | Low | 3.1 Yes.  3.2 Yes.  3.3 No.  Prospective study with interventions clearly defined. |
| Bias due to **deviations from intended interventions** | Low | 4.1 Probably no.  4.2 NA.  Variability in intervention, e.g. nutritional intervention for those at moderate-high risk of malnutrition in line with usual practice. |
| Bias due to **missing outcome data** (attrition bias) | Moderate | 5.1 No.  5.2 No.  5.3 No.  5.4 NA.  5.5 No.  Discharge data available from 58/60 who underwent cardiac rehabilitation. No patients lost to follow up. However outcome data only available for 76% of survivors followed up at second (18 month) follow up. |
| Bias in **measurement of the outcome** (detection bias) | Moderate | 6.1 Probably yes.  6.2 Yes.  6.3 NA.  6.4 No.  High variability in follow up time:  “patients underwent a follow-up program at our centre after 6-12 months (T1) and 18-24 months (T2).”  Variability also in measurement of outcome ie. some patients over telephone, some in person.  As it is a prospective study, subjective outcome measures are more at risk of bias due to unblended participants.  Some domains (e.g. measurement of prevalence of malnutrition) measured on admission but then not measured at discharge or in follow-up, so incomplete measurement of outcome associates with an exposure.  Unclear methodology surrounding time points or first and second follow up. (mean follow up time of 540 days provided but two follow up visits performed for most patients). |
| Bias in **selection of the reported result** (reporting bias) | Moderate | 7.1 Yes  7.2 No.  7.3 No.  EQ5D listed as outcome measure in the protocol, whereas in the study they instead reported EQVAS. |
| **Overall risk of bias judgement** | Serious |  |

| **Voller et al 2015 – Non-randomised retrospective observational study. (ROBINS-I)** | | |
| --- | --- | --- |
| **Type of Bias** | **Author’s Judgement (Low / Moderate / Serious / Critical)** | **Support for Judgement** |
| Bias arising from **confounding** | Serious | 1.1 Yes.  1.2 No.  1.3 NA.  1.4 No.  1.5 NA.  1.6 No.  1.7 NA.  1.8 NA.  With respect to assessing the degree of benefit this intervention has for the population of interest, one would expect a degree of improvement in exercise tolerance in a proportion of the patients with severe aortic stenosis in the weeks following valve replacement. This is a major confounding issue.  There was no control group of patients who had valve replacement but no cardiac rehabilitation, therefore flawed to attribute all improvement to cardiac rehabilitation.  With respect to using the sAVR patients as a “control group”, reasonable to use them as a benchmark for comparison of safety and adherence outcomes, however not a valid control group for assessing efficacy of intervention (cardiac rehab) as they have also received this.  Analysis showed significant baseline differences between the TAVI and SAVR groups. The authors made attempts to control for these variables through statistical analysis; however, there are likely to be other confounding variables that have not been controlled for. |
| Bias due to **selection of participants into the study** | Moderate | 2.1 No  2.2 NA.  2.3 NA.  2.4 Yes.  2.5 NA.  “Consecutive” TAVI/SAVR patients were selected.  However; following a discussion between reviewers, we felt the paper lacks pre-specified details on referral criteria for cardiac rehab (referral based on opinion of referring hospital clinical team) and there is, therefore, a risk of bias in the selection of patients to take part in CR following their TAVI or SAVR procedure. |
| Bias due to **classification of interventions** | Low | 3.1 Yes.  3.2 Yes.  3.3 No.  Low ambiguity in intervention groups and nature of intervention. |
| Bias due to **deviations from intended interventions** | Low | 4.1 No.  4.2 NA.  No reported cases of modification or discontinuation of intervention. |
| Bias due to **missing outcome data** (attrition bias) | Moderate | 5.1 No information.  5.2 No.  5.3 No.  5.4 NA.  5.5 NA.  Not specified whether the outcome data reported for all patients (seems unlikely that all 442 participants were able to complete cardiac rehabilitation and have all functional / emotional outcomes measured without complication). Possibility of only including patients who completed the rehab program and for whom outcomes were available (which would introduce selection bias). |
| Bias in **measurement of the outcome** (detection bias) | Low | 6.1 Probably no.  6.2 Yes.  6.3 Probably yes.  6.4 No.  Standardised method described in methods section.  Retrospective analysis, so participants and assessors unlikely to have knowledge of involvement in study at time measurements made. |
| Bias in **selection of the reported result** (reporting bias) | Moderate | 7.1 No.  7.2 No.  7.3 No.  No pre-specified plan/protocol available and as data collection and analysis was conducted retrospectively, authors could have chosen outcomes in the knowledge of whether any significant effect was demonstrated.  However, there is no evidence of selective reporting of outcomes and there is a broad range of outcomes reported with both positive and negative findings. |
| **Overall risk of bias judgement** | Serious |  |

| **Eichler et al 2017 - Non-randomised prospective observational study. (ROBINS-I).** | | |
| --- | --- | --- |
| **Type of Bias** | **Author’s Judgement (Low / Moderate / Serious / Critical)** | **Support for Judgement** |
| Bias arising from **confounding** | Serious | 1.1 Yes.  1.2 No.  1.3 NA.  1.4 No.  1.5 NA.  1.6 No.  1.7 NA.  1.8 NA.  With respect to assessing the degree of benefit this intervention has for the population of interest, one would expect a degree of improvement in exercise tolerance in a proportion of the patients with severe aortic stenosis in the weeks following valve replacement. This is a major confounding issue.  The authors themselves acknowledge this in this paper: “We do not consider the information about the clinical course of the TAVI procedure, which can have an influence on the dynamic of functional improvement.”  There was no control group of patients who had valve replacement but no cardiac rehabilitation, therefore flawed to attribute all improvement to cardiac rehabilitation. |
| Bias due to **selection of participants into the study** | Low | 2.1 No.  2.2 NA.  2.3 NA.  2.4 Yes.  2.5 NA.  Clearly depicted flow chart of inclusion process. All patients undergoing CR after TAVI at participating CR centres included. All patients followed up at CR admission and discharge. |
| Bias due to **classification of interventions** | Low | 3.1 Yes.  3.2 No.  3.3 No.  Interventions clearly defined with a low risk of ambiguity. |
| Bias due to **deviations from intended interventions** | Low | 4.1 No.  4.2 NA.  No reported deviations |
| Bias due to **missing outcome data** (attrition bias) | Moderate | 5.1 No.  5.2 No.  5.3 No.  5.4 NA.  5.5 No.  Missing data for some outcome measures e.g. exercise capacity only measured in 78/136 participants. |
| Bias in **measurement of the outcome** (detection bias) | Moderate | 6.1 Probably yes.  6.2 Yes.  6.3 NA.  6.4 Yes  As it is a prospective study, subjective outcome measures are more at risk of bias due to unblended participants.  Outcome assessor unspecified for 6MWT.  Only 78/136 patients contributing towards exercise capacity measurement – suggests systematic errors in measurement of the outcome related to intervention received. |
| Bias in **selection of the reported result** (reporting bias) | Low | 7.1 No.  7.2 No.  7.3 No. |
| **Overall risk of bias judgement** | Serious |  |

| **Penati et al 2021 – Non-randomised observational study. Study design not specified as retrospective or prospective. (ROBINS-I)** | | |
| --- | --- | --- |
| **Type of Bias** | **Author’s Judgement (Low / Moderate / Serious / Critical)** | **Support for Judgement** |
| Bias arising from **confounding** | Serious | 1.1 Yes.  1.2 No.  1.3 NA.  1.4 No.  1.5 NA.  1.6 No.  1.7 NA.  1.8 NA.  With respect to assessing the degree of benefit this intervention has for the population of interest, one would expect a degree of improvement in exercise tolerance in a proportion of the patients with severe aortic stenosis in the weeks following valve replacement. This is a major confounding issue.  There was no control group of patients who had valve replacement but no cardiac rehabilitation, therefore flawed to attribute all improvement to cardiac rehabilitation. |
| Bias due to **selection of participants into the study** | Serious | 2.1 No information.  2.2 No information.  2.3 No information.  2.4 No information.  2.5 Probably no.  No specification as to retrospective vs observational study design. No inclusion criteria provided or selection method given, not clear whether patients were consecutive.  “The study considered 48 Caucasian patients following TAVI who underwent a residential CR program at the Complex Operational Unit of Cardiac/Pulmonary Rehabilitation of the ASST Pini-CTO Hospital between 01 January 2019 and 31 December 2019.” This statement suggests selection based on ethnicity +/- other factors. |
| Bias due to **classification of interventions** | Serious | 3.1 No.  3.2 No information.  3.3 No information.  Unclear if study retrospective or prospective. High variability within intervention group:  “Half of them were assessed using the SPPB scale [14] and assigned to an aerobic training of deambulatory type with intermittent mode, alternating average repetitions of 250 meters with average breaks of 3 min. The remaining patients were assessed by means of a 6MWT [15] and assigned to continuous aerobic training with the cycle ergometer”  Different intervention applied to these two groups however results not interpreted as two separate groups. |
| Bias due to **deviations from intended interventions** | Low | 4.1 No.  4.2 NA.  No deviations from intended interventions reported. |
| Bias due to **missing outcome data** (attrition bias) | Serious | 5.1 No.  5.2 No information.  5.3 Probably yes.  5.4 NA.  5.5 No.  SPPB done in half, 6MWT done in half, Barthel done in all. Unclear if this was an intentional decision as part of study design or as a result of an extrinsic factor. Telephone follow up only done in 30/49 participants. The patient that resigned was excluded from analysis. |
| Bias in **measurement of the outcome** (detection bias) | Serious | 6.1 Yes.  6.2 Yes.  6.3 Yes.  6.4 No.  Completing questionnaires by telephone vulnerable to detection bias as assessors unblinded to intervention received. They performed telephone assessments of subjective outcomes reported such as “state of health” without a scoring system/index used, and “continuing aerobic activity” but no definition as to what would comprise minimum level of aerobic activity to be considered yes for this. |
| Bias in **selection of the reported result** (reporting bias) | Critical | 7.1 Yes.  7.2 No.  7.3 No.  No prespecified plan or protocol. BMI and biochemical parameters such as Hb and Albumin misleadingly reported graphically but not discussed in method or results sections.  Unclear how many patients were included in each outcome measure group. |
| **Overall risk of bias judgement** | Critical | Significant flaws in methodology of this paper and poor level of detail regarding methodology and study design. Opinion of researcher that this study is too problematic to provide any useful evidence and should not be included in any synthesis. |

| **Yu et al 2021 – Non-randomised, retrospective single-arm observational study. (ROBINS-I)** | | |
| --- | --- | --- |
| **Type of Bias** | **Author’s Judgement (Low / Moderate / Serious / Critical)** | **Support for Judgement** |
| Bias arising from **confounding** | Serious | 1.1 Yes.  1.2 No.  1.3 NA.  1.4 No.  1.5 NA.  1.6 No.  1.7 NA.  1.8 NA.  With respect to assessing the degree of benefit this intervention has for the population of interest, one would expect a degree of improvement in exercise tolerance in a proportion of the patients with severe aortic stenosis in the weeks following valve replacement. This is a major confounding issue.  There was no control group of patients who had valve replacement but no cardiac rehabilitation, therefore flawed to attribute all improvement to cardiac rehabilitation. |
| Bias due to **selection of participants into the study** | Moderate | 2.1 Yes.  2.2 NA.  2.3 NA.  2.4 Probably yes.  2.5 NA.  “One hundred and eight consecutive patients scheduled for TAVI … were evaluated before TAVI. One hundred patients received TAVI successfully. Of them, 90 patients completed the CGA-based CR. Sixty-nine and 75  patients underwent six-minute walk test (6MWT) before discharge and after a month, respectively”  Inclusion dependent on completion of cardiac rehabilitation rather than enrolment. |
| Bias due to **classification of interventions** | Low | 3.1 Probably yes.  3.2 Yes.  Retrospective observational study. No comparison group. Intervention group = those who completed CGA-guided CR following TAVI. |
| Bias due to **deviations from intended interventions** | Low | 4.1 No.  4.2 NA.  No apparent deviations from intended interventions. |
| Bias due to **missing outcome data** (attrition bias) | Moderate | 5.1 Probably no.  5.2 No.  5.3 No.  5.4 NA.  5.5 Probably no.  6MWT assessed in 69 patients ‘before discharge after TAVI’ and 75 patients ‘after CR.’ CGA assessed in 90 patients.  No data given on how many patients were included in analysis for each of the CGA domains e.g. MMSE, HADS-A etc. |
| Bias in **measurement of the outcome** (detection bias) | Low | 6.1 Probably no.  6.2 Probably no.  6.3 Yes.  6.4 No.  Some patient reported outcomes however standardised tools (e.g. HADS) used. |
| Bias in **selection of the reported result** (reporting bias) | Moderate | 7.1 No.  7.2 No.  7.3 No.  No pre-specified plan/protocol available. |
| **Overall risk of bias judgement** | Serious |  |

| **Kleczynski et al 2021 – Non-randomised, observational study. (ROBINS-I)** | | |
| --- | --- | --- |
| **Type of Bias** | **Author’s Judgement (Low / Moderate / Serious / Critical)** | **Support for Judgement** |
| Bias arising from **confounding** | Moderate | 1.1 Yes.  1.2 No.  1.3 NA.  1.4 Probably no.  1.5 NA.  1.6 No.  1.7 NA.  1.8 NA.  Populations were well matched for clinical characteristics and baseline 5MWT and 6MWD, however (As acknowledged by authors) likely to be fundamental socioeconomic and behavioural differences between population who decline and population who accept rehabilitation:  “Patients who decline CR are different from those willing to participate, not in clinical characteristics but in behavioral or socioeconomical aspects, which may play an important role” |
| Bias due to **selection of participants into the study** | Moderate | 2.1 Probably yes.  2.2 Probably yes.  2.3 Probably no.  2.4 Yes.  2.5 Probably no.  “We included 105 consecutive patients”. Participants reported as having been consecutively chosen; however, unless rates of uptake of cardiac rehabilitation truly are 50%, unclear how equal sample sizes chosen.  Also unclear whether only patients who had completed the full period of inpatient cardiac rehabilitation were included as part of inclusion criteria for the study (seems unlikely that 105 consecutive patients all completed full period with no missing data.) |
| Bias due to **classification of interventions** | Low | 3.1 Yes.  3.2 Yes.  3.3 No.  Intervention (Cardiac rehabilitation) vs control (Discharged home, having declined cardiac rehabilitation). Clearly defined groups, not ambiguous. |
| Bias due to **deviations from intended interventions** | Low | 4.1 No.  4.2 NA  No indication of non-adherence to intervention (cardiac rehabilitation) by trial participants; unclear whether only patients who had completed the full period of inpatient cardiac rehabilitation were included as part of inclusion criteria for the study (seems unlikely that 105 consecutive patients all completed full period with no missing data.) |
| Bias due to **missing outcome data** (attrition bias) | Moderate | 5.1 Probably no.  5.2 No.  5.3 Probably no.  5.4 Probably yes.  5.5 No.  Apparently complete datasets for patients at 30 day, 6 month and 12 month follow up presented, with the same number of patients in the group as at baseline despite stating in the results all-cause death rates were similar “(7.7% in the CR group vs 7.5% in the DH group, p = 0.91)”. Therefore number of patients contributing to each follow up result has not been accurately reported or the information has not been reported clearly. |
| Bias in **measurement of the outcome** (detection bias) | Moderate | 6.1 Probably yes.  6.2 Yes.  6.3 Yes.  6.4 No.  Outcome measure (especially of patient-reported outcome measures) could have been influenced, however unclear to what degree patients were aware they were being compared to a control/intervention group. |
| Bias in **selection of the reported result** (reporting bias) | Low | 7.1 No.  7.2 No.  7.3 No. |
| **Overall risk of bias judgement** | Moderate |  |

| **Butter et al, 2018 – Non-randomised, retrospective observational study. (ROBINS-I)** | | |
| --- | --- | --- |
| **Type of Bias** | **Author’s Judgement (Low / Moderate / Serious / Critical)** | **Support for Judgement** |
| Bias arising from **confounding** | Serious | 1.1 Yes.  1.2 No.  1.3 NA.  1.4 Probably no.  1.5 NA  1.6 No.  1.7 NA.  1.8 NA.  With respect to assessing the degree of benefit this intervention has for the population of interest, one would expect a degree of improvement in exercise tolerance in a proportion of the patients with severe aortic stenosis in the weeks following valve replacement. This is a major confounding issue.  With respect to benefit of Cardiac over Geriatric rehabilitation: baseline variables affecting likelihood of patient being referred for CR vs GR will influence mortality outcomes. GR population by definition “have lower physical, mental, cognitive, and psychologic capacities.”  The authors also acknowledge possibility of bias from confounding due to uncontrollable socioeconomic factors:  “Patients declining rehabilitation may have been behaviourally and socio-economically different from those who chose to perform rehabilitation.” |
| Bias due to **selection of participants into the study** | Low | 2.1 No.  2.2 NA.  2.3 NA.  2.4 Yes.  2.5 NA.  Eligibility and enrolment of participants clearly defined: “all patients undergoing elective TAVI at the Brandenburg Heart Centre in Bernau, Germany between 2008 and 2016 were enrolled and prospectively followed by telephone calls or letters. Subjects were excluded if they died during initial hospital stay.” Prospective study design. |
| Bias due to **classification of interventions** | Low | 3.1 Yes.  3.2 Yes.  3.3 No.  Interventions clearly defined, unambiguous classification. |
| Bias due to **deviations from intended interventions** | Low | 4.1 No.  4.2 NA  No reported deviations. “In accordance with standard clinical practice in Germany, all patients were offered rehabilitation after the TAVI procedure in preparation for hospital discharge.” |
| Bias due to **missing outcome data** (attrition bias) | Moderate | 5.1 No.  5.2 No.  5.3 Yes  5.4 Probably yes.  5.5 Probably no.  Significant loss to follow up (77/366 in control “no rehabilitation” group and 113/651 in the rehabilitation group) |
| Bias in **measurement of the outcome** (detection bias) | Low | 6.1 Probably no.  6.2 Yes.  6.3 Yes.  6.4 No.  Main outcome is objective. |
| Bias in **selection of the reported result** (reporting bias) | Low | 7.1 No.  7.2 No.  7.3 No.  Whilst 2 different subgroups have been combined to generate overall effect estimate of rehabilitation vs no rehabilitation, breakdown of these subgroups available. |
| **Overall risk of bias judgement** | Serious |  |

| **Myszenski et al 2021 – Non-randomised retrospective study with historical control. (ROBINS-I)** | | |
| --- | --- | --- |
| **Type of Bias** | **Author’s Judgement (Low / Moderate / Serious / Critical)** |  |
| Bias arising from **confounding** | Serious | 1.1 Yes.  1.2 No.  1.3 NA.  1.4 No.  1.5 NA.  1.6 No.  1.7 NA.  1.8 NA.  Univariate 2-group comparisons performed; however, this will not have controlled for residual confounding. The authors acknowledge potential bias through confounding relating to socioeconomic and other factors:  “The patient’s level of function prior to cardiac surgery...level of social support…not accounted for”.  Statistically significant differences in baseline characteristics noted for ‘heart failure prior 2 wk” and “5m walk test prior”.  Relatively long time period of data collection (March 2012 – Dec 2015) during which one would expect there to be advances in procedure technique (though note that patients who experienced major complications were excluded from this study), greater experience of the staff with respect to post-procedural care. |
| Bias due to **selection of participants into the study** | Serious | 2.1 Yes.  2.2 No information.  2.3 No information.  2.4 Yes.  Exclusion criteria included “..the occurrence of major events including death, stroke, myocardial infarction (MI), additional cardiac or vascular surgery, electrophysiological monitoring, pacemaker placement, atrial fibrillation, other cardiovascular repair or surgery required, gastrointestinal bleeds, hematoma or access bleeds, or complications”. Unreported as to how many of these patients have been excluded. This study therefore is only assessing the efficacy of the intervention to patients with an entirely uncomplicated post-procedural course.  It may under-estimate any negative or dangerous complications associated with the intervention (e.g. if incidence of post-procedural MI higher in group receiving early physical therapy, for example). |
| Bias due to **classification of interventions** | Low | 3.1 Yes.  3.2 Yes.  3.3 No.  Time cut-off used (pre and post Nov 2^nd^ 2014) so no ambiguity. |
| Bias due to **deviations from intended interventions** | Low | 4.1 No.  4.2 NA.  No reported deviations. |
| Bias due to **missing outcome data** (attrition bias) | Low | 5.1 Yes.  5.2 No.  5.3 No.  5.4 NA.  5.5 NA.  No reported missing data (hard outcomes measured at point of discharge from hospital). |
| Bias in **measurement of the outcome** (detection bias) | Low | 6.1 Probably no.  6.2 Yes.  6.3 Yes.  6.4 No.  Retrospective study but objective measures used. |
| Bias in **selection of the reported result** (reporting bias) | Moderate | 7.1 No.  7.2 No.  7.3 No.  Due to retrospective study design, outcomes may have been selected for their results. No pre-existing protocol/analysis plan. |
| **Overall risk of bias judgement** | Serious |  |

| **Hart et al, 2021 – Controlled before and after study (Adapted ROBINS-I)** | | |
| --- | --- | --- |
| **Type of Bias** | **Author’s Judgement (Low Risk/High Risk/Some Concerns)** | **Support for Judgement** |
| Bias arising from **confounding** | Serious | 1.1 Yes.  1.2 No.  1.3 NA.  1.4 No.  1.5 NA.  1.6 No.  1.7 NA.  1.8 NA.  Authors acknowledge differences in baseline characteristics e.g. that “the post-intervention group had more patients admitted electively for TAVR”. Age distribution of pre-intervention group and post-intervention group not reported; this could potentially represent a major confounding factor.  COVID-19 will have affected the post-intervention group but not the pre-intervention group. E.g. follow up telephone interviews in post-intervention group. But need to acknowledge also the hugely significant effect and pressure Covid would have had on services during the time of the pre-intervention group. There is, therefore, a significant risk of confounding in this group. |
| Bias due to **selection of participants into the study** | Moderate | 2.1 Probably no.  2.2 NA.  2.3 NA.  2.4 Yes.  2.5 NA.  Consecutive recruitment of all eligible patients within a given time period. Exclusion criteria clearly specified, which would likely be known prior to implementing intervention: “Patients who could not be successfully extubated in the operating room, experienced a major vascular complication or a major stroke after the procedure, or died were excluded.”  However, there is ambiguity in eligibility criteria for intervention:  “Of these 46 patients, 38 were deemed good candidates for the specialized order set and early mobility protocol and formed the postintervention group”  Criteria for what “deems” a patient a good candidate not clear; not clear whether the 8 excluded patients all fall into the categories above. |
| Bias due to **classification of interventions** | Low | 3.1 Yes.  3.2 Yes.  3.3 No.  Intervention clearly defined (application of a protocol). |
| Bias due to **deviations from intended interventions** | Moderate | 4.1 Yes.  4.2 Yes.  Unblinded prospective study with key stakeholders likely to be invested in application of the protocol/intervention. As a result, potential to over-estimate effect of intervention if those implementing intervention are aware of its success being measured e.g. expediting discharges.  COVID-19 will have affected the post-intervention group but not the pre-intervention group. E.g. completing questionnaires/interviews over telephone in post-intervention group. |
| Bias due to **missing outcome data** (attrition bias) | Moderate | 5.1 Probably yes.  5.2 No information.  5.3 No information.  5.4 Na.  5.5 No information.  Study appears to present full dataset for 38 participants in each group, however authors do not specify that no participants lost to follow up. If participants excluded due to missing data then this would lead to selection bias. |
| Bias in **measurement of the outcome** (detection bias) | Moderate | 6.1 Probably yes.  6.2 Yes.  6.3 No.  6.4 No.  Pre-intervention data set was collected retrospectively whereas post-intervention data was collected prospectively, with assessors and participants aware of this intervention that they had received. This could potentially have influenced the more subjective outcomes such as KCCQ and influenced decision-making surrounding discharge.  In second group some KCCQs had to be completed over the phone due to COVID-19 pandemic; different method of outcome assessment however this is unlikely to have had significant impact. |
| Bias in **selection of the reported result** (reporting bias) | Low | 7.1 No.  7.2 No.  7.3 No.  Appropriate reporting of all outcomes mentioned in the pre-specified aims. |
| **Overall risk of bias judgement** | Serious |  |

| **Brocki et al. 2023** | | |
| --- | --- | --- |
| **Type of Bias** | **Author’s Judgement (Low Risk/High Risk/Some Concerns)** | **Support for Judgement** |
| Bias arising from **confounding** | Serious | - 1. Yes.   2. No.   3. NA.   4. No.   5. NA.   6. No.   7. No.   8. NA.   The authors acknowledge that the TAVI itself is a significant confounder. They stated “since we had no control group, we cannot exclude a natural improvement in outcomes due to benefits from the surgery”. They seek to conduct an RCT to address this issue. |
| Bias due to **selection of participants into the study** | Serious | 2.1 Yes.  2.2 Yes.  2.3 NA.  2.4 No information.  2 participants were excluded after enrolment as they were not able to use the electronic tablet that was required to complete the intervention. |
| Bias due to **classification of interventions** | Low | 3.1 Yes.  3.2 Yes.  3.3 No. |
| Bias due to **deviations from intended interventions** | Moderate | 4.1 No.  4.2 NA.  4.3 No information.  4.4 Probably yes.  4.5 Probably yes.  4.6 NA.  The authors state that “Six out of seven [participants] reached the 60% pre-established success rate for adherence to the web-based supervised training sessions” |
| Bias due to **missing outcome data** (attrition bias) | Serious | 5.1 No.  5.2 Probably no.  5.3 Probably yes.  5.4 No information.  5.5 Probably no.  47% retainment rate. Single-arm study so not able to determine if the amount of missing data was similar across interventions. |
| Bias in **measurement of the outcome** (detection bias) | Moderate | 6.1 Yes.  6.2 No information.  6.3 No information.  Single-arm study. |
| Bias in **selection of the reported result** (reporting bias) | Low | 7.1 No.  7.2 No.  7.3 No. |
| **Overall risk of bias judgement** | Serious |  |

| **Frank et al. 2024** | | |
| --- | --- | --- |
| **Type of Bias** | **Author’s Judgement (Low Risk/High Risk/Some Concerns)** | **Support for Judgement** |
| Bias arising from **confounding** | Moderate | - 1. Probably yes.   2. No.   3. NA.   4. Probably yes.   5. Yes.   6. No information.   7. Probably yes.   8. Probably yes.   Intervention arm compared with a standard care arm. This should help to control for the impact of receiving the TAVI procedure. However, it introduces a temporal element as the standard-care arm consisted of data retrospectively collected prior to the implementation of the intervention (BENCHMARK). It may be that other practices had improved during that period.  It also noted that although for most outcome measures they compared the post-BENCHMARK implementation data with pre-intervention data, in the case of quality of life, they compared the pre- and post- TAVI results within the post-intervention participants. |
| Bias due to **selection of participants into the study** | Low | 2.1 No.  2.2 NA.  2.3 NA.  2.4 Yes.  2.5 NA. |
| Bias due to **classification of interventions** | Low | 3.1 Probably yes.  3.2 Yes.  3.3 No.  Intervention group is well defined with a clear inclusion and exclusion criteria. |
| Bias due to **deviations from intended interventions** | Low | 4.1 Probably no.  4.2 NA.  4.3 Probably yes.  4.4 Yes.  4.5 Probably yes.  4.6 NA. |
| Bias due to **missing outcome data** (attrition bias) | Serious | 5.1 No.  5.2 Yes.  5.3 No information.  5.4 NA.  5.5 NA.  The authors stated that pre-intervention patients were excluded from retrospective analysis if “key variables for the assessment were missing”. However they did not report how many participants had missing data. They stated that the data was not imputed but make no other reference to missing data. |
| Bias in **measurement of the outcome** (detection bias) | Low | 6.1 Probably no.  6.2 No information.  6.3 Probably yes.  6.4 Probably no.  There was clearly defined method for measuring the primary outcome, which was objective. |
| Bias in **selection of the reported result** (reporting bias) | Low | 7.1 No.  7.2 No.  7.3 No.  Appropriate reporting of all outcomes mentioned in the pre-specified aims. |
| **Overall risk of bias judgement** | Serious |  |

| **Schwesinger et al. 2024** | | |
| --- | --- | --- |
| **Type of Bias** | **Author’s Judgement (Low Risk/High Risk/Some Concerns)** | **Support for Judgement** |
| Bias arising from **confounding** | Serious | - 1. Probably yes.   2. No.   3. NA.   4. Probably yes.   5. Probably yes.   6. No information.   7. Probably yes.   8. Probably yes.   Although this study had a comparator arm, the patients who were within this arm may have represented a different population of patients. The baseline characteristics are not significantly different however this is from the select characteristics that have been measured. The authors attempted to adjust for potential confounders (age, sex, BMI, the number of medications) |
| Bias due to **selection of participants into the study** | Low | 2.1 No.  2.2 NA.  2.3 NA.  2.4 Probably yes.  2.5 NA.  Participants appropriately selected. |
| Bias due to **classification of interventions** | Low | 3.1 Yes.  3.2 Yes.  3.3 No.  Intervention clearly defined. Not possible to change the intervention status after the outcome has been measured. |
| Bias due to **deviations from intended interventions** | Low | 4.1 Probably no.  4.2 NA.  4.3 Probably yes.  4.4 No information.  4.5 No information.  4.6 NA. |
| Bias due to **missing outcome data** (attrition bias) | Moderate | 5.1 No information.  5.2 Probably no.  5.3 Probably no.  5.4 NA.  5.5 NA.  There was no information about how many participants completed the study. |
| Bias in **measurement of the outcome** (detection bias) | Low | 6.1 Probably no.  6.2 No information.  6.3 Yes.  6.4 Probably no.  Assessors not blinded but unlikely to influence the outcome of post-operative delirium as there was a clearly defined and standardised assessment tool. |
| Bias in **selection of the reported result** (reporting bias) | Low | 7.1 No.  7.2 No.  7.3 No.  Appropriate reporting of all outcomes mentioned in the pre-specified aims. |
| **Overall risk of bias judgement** | Serious |  |
