# Supplementary material for: Comprehensive geriatric assessment, and related interventions, to improve outcomes for older patients undergoing transcatheter aortic valve implantation (TAVI): a systematic review
Source: Eur Geriatr Med. 2024 Sep 27;15(6):1615–30. doi: 10.1007/s41999-024-01035-5 (PMC11631815; doi:10.1007/s41999-024-01035-5)
Supplement: Supplementary file 5 — Supplementary file5 (DOCX 17 kb) [file 41999_2024_1035_MOESM5_ESM.docx]

**CGA in older adults undergoing TAVI**

| **Certainty assessment** | | | | | | | **Certainty** |
| --- | --- | --- | --- | --- | --- | --- | --- |
| **№ of studies** | **Study design** | **Risk of bias** | **Inconsistency** | **Indirectness** | **Imprecision** | **Other considerations** |  |
| **Functional independence** | | | | | | | |
| 2 | Non-randomised studies | serious^a^ | serious^b,c^ | not serious | serious^d^ | none | ⨁◯◯◯ Very low |

#### Explanations

a. Risk of bias downgraded due to a serious risk of confounding

b. Heterogeneity of populations, outcome measures and outcomes.

c. 1 study showed a statistically significant positive effect on functional independence. 1 study did not show a statistically significant impact on functional independence.

d. The 95% confidence intervals are narrow but overlap between the before and after results.

**Multi-component interventions in older adults undergoing TAVI**

| **Certainty assessment** | | | | | | | | **Certainty** |
| --- | --- | --- | --- | --- | --- | --- | --- | --- |
| **№ of studies** | **Study design** | **Risk of bias** | **Inconsistency** | **Indirectness** | **Imprecision** | | **Other considerations** |  |
| **Functional independence** | | | | | |  |  |  |
| 5 | non-randomised studies | very serious^a^ | serious^b,c^ | serious^d^ | serious^e^ | | none | ⨁◯◯◯ Very low |
| **Quality of life** | | | | | |  |  |  |
| 4 | non-randomised studies | very serious^a^ | serious^b,f^ | serious^d^ | serious^e^ | | none | ⨁◯◯◯ Very low |
| **Mortality** | | | | | |  |  |  |
| 1 | non-randomised studies | very serious^a^ | not serious | serious^g^ | not serious | | none | ⨁◯◯◯ Very low |

#### Explanations

a. Serious risk of confounding.

b. Heterogeneity of populations, interventions, outcome measures and outcomes.

c. 3 of the 5 studies showed significant improvements. 2 of the 5 showed non-significant results.

d. Indirectness of the intervention and comparator.

e. Wide 95% confidence intervals. Total sample size small.

f. 3 of the 4 studies showed significant improvements. 1 of the 4 showed non-significant results.

g. Indirectness of the intervention - may not be replicable in wider healthcare setting.

**The use of single-component interventions in older adults undergoing TAVI**

| **Certainty assessment** | | | | | | | | | **Certainty** |
| --- | --- | --- | --- | --- | --- | --- | --- | --- | --- |
| **№ of studies** | **Study design** | **Risk of bias** | **Inconsistency** | **Indirectness** | **Imprecision** | | | **Other considerations** |  |
| **Functional independence** | | | | | | |  |  |  |
| 2 | non-randomised studies | very serious^a^ | serious^b,c^ | serious^d^ | serious^e^ | | | none | ⨁◯◯◯ Very low |
| **Functional independence** | | | | | | |  |  |  |
| 1 | randomised trials | serious^f^ | not serious | not serious | serious^e^ | | | none | ⨁⨁◯◯ Low |
| **Quality of life** | | | | | | |  |  |  |
| 6 | randomised trials | very serious^g^ | serious^b,h^ | serious^i^ | serious^e^ | | | none | ⨁◯◯◯ Very low |
| **Quality of life** | | | | | |  |  |  |  |
| 1 | non-randomised studies | very serious^a^ | not serious | not serious | serious^e^ | | | none | ⨁◯◯◯ Very low |
| **Mortality** | | | | | |  |  |  |  |
| 2 | randomised trials | serious^j^ | not serious | serious^k^ | serious^l^ | | | none | ⨁◯◯◯ Very low |
| **Mortality** | | | | | |  |  |  |  |
| 1 | non-randomised studies | serious^m^ | not serious | serious^n^ | serious^o^ | | | none | ⨁◯◯◯ Very low |

#### Explanations

a. Serious risk of confounding.

b. Heterogeneity of interventions, outcome measures and outcomes.

c. 1 study showed a statistically significant improvement in functional independence. 1 study did not show a statistically significant change.

d. Indirectness of the intervention, comparator and outcome measure.

e. Wide 95% confidence intervals. Total sample size small.

f. Moderate risk of bias due to difference in baseline characteristics following randomisation, deviation from the intended intervention, missing outcome data, not all outcome data being reported as per the trial protocol.

g. Missing outcome data and deviations from the intervention protocol.

h. 5 of 6 studies did not show a significant improvement. 1 study showed a showed a significant improvement in quality of life.

i. Indirectness of the intervention

j. 1 study was non-blinded study. There is no study protocol available so there was not enough information available to ascertain whether all the measured outcomes were reported.

k. Indirectness of the intervention (one study intervention group included 2 weeks of individual, daily physiotherapy exercise, the other study had an intervention room consisting of early mobilisation post procedure)

l. Underpowered study. Sample size required calculated to be 220, 108 recruited.

m. Serious risk of attrition bias.

n. Indirectness of some of the outcome measures.

o. Wide interquartile ranges.
